# Disruption of mitochondrial dynamics triggers muscle inflammation through interorganellar contacts and mitochondrial DNA mislocation

**DOI:** 10.1038/s41467-022-35732-1

**Published:** 2023-01-06

**Authors:** Andrea Irazoki, Isabel Gordaliza-Alaguero, Emma Frank, Nikolaos Nikiforos Giakoumakis, Jordi Seco, Manuel Palacín, Anna Gumà, Lykke Sylow, David Sebastián, Antonio Zorzano

**Affiliations:** 1 Institute for Research in Biomedicine (IRB Barcelona), The Barcelona Institute of Science and Technology, Baldiri Reixac, 10-12, Barcelona, Spain; 2 Departament de Bioquímica i Biomedicina Molecular, Facultat de Biologia, Universitat de Barcelona, Barcelona, Spain; 3 Centro de Investigación Biomédica en Red de Diabetes y Enfermedades Metabólicas Asociadas (CIBERDEM) Instituto de Salud Carlos III, Madrid, Spain; 4 Department of Biomedical Sciences (BMI), Faculty of Health and Medical Sciences, University of Copenhagen, Copenhagen, Denmark; 5 CIBER de Enfermedades Raras (CIBERER), Instituto de Salud Carlos III, Madrid, Spain; 6 Institute of Biomedicine of the University of Barcelona (IBUB), Barcelona, Spain; 7 Department of Nutrition, Exercise and Sports, Faculty of Science, University of Copenhagen, Copenhagen, Denmark; 8 Department of Biochemistry and Physiology, Faculty of Pharmacy and Food Sciences, University of Barcelona, Barcelona, Spain

**Keywords:** Mitochondria, Skeletal muscle, Mitochondria

## Abstract

Some forms of mitochondrial dysfunction induce sterile inflammation through mitochondrial DNA recognition by intracellular DNA sensors. However, the involvement of mitochondrial dynamics in mitigating such processes and their impact on muscle fitness remain unaddressed. Here we report that opposite mitochondrial morphologies induce distinct inflammatory signatures, caused by differential activation of DNA sensors TLR9 or cGAS. In the context of mitochondrial fragmentation, we demonstrate that mitochondria-endosome contacts mediated by the endosomal protein Rab5C are required in TLR9 activation in cells. Skeletal muscle mitochondrial fragmentation promotes TLR9-dependent inflammation, muscle atrophy, reduced physical performance and enhanced IL6 response to exercise, which improved upon chronic anti-inflammatory treatment. Taken together, our data demonstrate that mitochondrial dynamics is key in preventing sterile inflammatory responses, which precede the development of muscle atrophy and impaired physical performance. Thus, we propose the targeting of mitochondrial dynamics as an approach to treating disorders characterized by chronic inflammation and mitochondrial dysfunction.

## Abstract (web summary)

Some forms of mitochondrial dysfunction can cause sterile inflammation, but the way in which it might affect muscle fitness is not well understood. Here, the authors show that altered mitochondrial dynamics can cause the production of mitochondrial DNA-driven inflammatory signals mediated by endosome-mitochondria contacts, leading to muscle inflammation, atrophy, reduced physical performance and enhanced exercise-induced inflammatory responses.

## Introduction

Mitochondrial dynamics refers to changes in the morphology of mitochondria and the movement of these organelles, allowing cells to respond to environmental stimuli. The transition from fused to fragmented mitochondria, and vice versa, is governed by GTPases that form the core machinery of mitochondrial dynamics. Mitofusins 1 and 2 (MFN1/2), along with OPA1, are responsible for mitochondrial fusion, whereas DRP1, FIS1, MFF, and MID49/51 promote mitochondrial fission, DRP1 being the key trigger of this process. Maintenance of an optimal balance between mitochondrial fusion and fission, which ensures mitochondrial, and hence cellular, homeostasis, requires the correct expression and activity of all these proteins^1^. Indeed, ablation of MFN1 or 2 in a range of tissues, including muscle^2–7^, liver^8–10^, adipose tissue^11,12^, neurons^13–15^, and immune cells^16^, promotes changes in glucose metabolism, insulin sensitivity, altered thermogenesis, growth defects, tissue atrophy, accelerated aging, and systemic inflammation.

Inflammation is a physiological response to harmful stimuli. Cellular inflammatory responses are induced by the recognition of damaged- or pattern-associated molecular patterns (DAMPs or PAMPs), among which mitochondrial DNA (mtDNA) is considered to play a major role^17^. Given that intracellular DNA sensors can recognize mtDNA and engage cognate inflammatory pathways, the mislocation of mtDNA outside of mitochondria is a key factor in triggering sterile inflammation. In fact, several forms of mitochondrial dysfunction have been associated with mtDNA mislocation-mediated inflammatory signals^6,18–23^, yet, whether imbalanced mitochondrial dynamics are also associated with similar processes remains unknown. Regarding mtDNA-induced inflammation, endosomal localization of mtDNA can induce the toll-like receptor 9 (TLR9)-dependent nuclear factor kappa B (NFκB) inflammatory pathway^24^, and cytosolic mtDNA can engage with either cyclic GAMP synthase (cGAS)^20^ or inflammasomes^25,26^. cGAS activation triggers the type I interferon (IFN) response and the NFκB -dependent pathway, whereas the activation and assembly of inflammasomes promote the processing and secretion of caspase 1-mediated interleukin 1β and 18.

The plethora of intracellular inflammatory pathways responsive to mislocated mtDNA sequences reveals the relevance of maintaining mitochondrial homeostasis. In fact, associations between mitochondrial stress and mtDNA-dependent inflammation in non-immune cells have been reported upon downregulation of several mitochondrial proteins, including PINK1^22^, FUNDC1^21^, BNIP3^19^, IRGM1^23^ OPA1^6^, YME1L^18^, and TFAM^20^. The activation of inflammation upon depletion of the mitophagy proteins PINK1, FUNDC1, BNIP3, or IRGM1 suggests that mitophagy plays a key role in preventing mtDNA mislocation. In addition, depletion of TFAM is linked to mtDNA instability, which would explain mtDNA mislocation and consequent inflammation. In this regard, the coupling of OPA1 depletion to inflammation suggests that mitochondrial dynamics is a key process in the prevention of mtDNA-dependent inflammation. However, whether the activation of inflammation in this context is a consequence of imbalanced mitochondrial dynamics or driven by intrinsic functions of OPA1 was unanswered. Thus, given the pivotal role played by mitochondrial dynamics in the regulation of mitochondrial homeostasis, addressing the implication of mitochondrial dynamics in the activation of sterile inflammation required attention. Also, in the specific context of muscle biology, whether muscle inflammation precedes muscle atrophy or whether it is triggered by this process of muscle loss is not clearly addressed yet.

Here we show that perturbations in mitochondrial dynamics initiate intracellular inflammatory responses associated with mtDNA mislocation in skeletal muscle cells, and that the inflammatory profiles observed are dependent on mitochondrial morphology. Focusing on the mechanism linking mitochondrial fragmentation to inflammation, we demonstrate that the downregulation of Mfn1 promotes physical approximation of mitochondria to Rab5^+^ vesicles or early endosomes, mediated by a newly discovered interaction between Mfn2 and Rab5C, which is a requirement for mtDNA-TLR9 interaction and NFκB-mediated inflammation. Furthermore, skeletal muscle-specific Mfn1 knockout mice recapitulate the inflammatory phenotype observed in cells and present muscle atrophy, compromised physical performance, and an enhanced exercise-induced systemic inflammatory response characterized by IL6 upregulation. Chronic anti-inflammatory treatment ameliorates muscle atrophy, and, consequently, physical capacity and the exercise-induced IL6 response. These observations thus indicate that inflammation plays a causative role in the development of atrophy and its complications. Collectively, we demonstrate that imbalances in mitochondrial dynamics promote mtDNA mislocation, triggering inflammation in a mitochondrial morphology-dependent manner, and we characterize the precise molecular linkers between mitochondrial fragmentation and inflammation. Furthermore, we assess the impact of this molecular pathway in vivo and reveal that muscle inflammation driven by mitochondrial fragmentation is key in the development of muscle atrophy, reduced muscle function, and exacerbated exercise-induced responses.

## Results

### Altered mitochondrial dynamics triggers mtDNA-dependent sterile inflammation

**Fig. 1:**
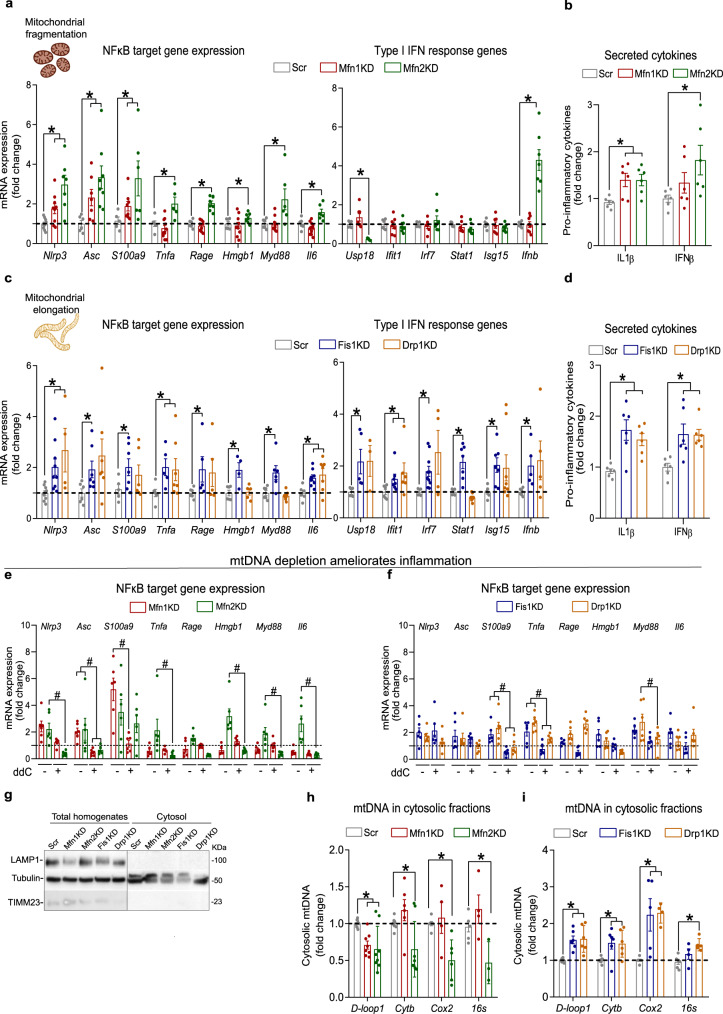
Opposite mitochondrial morphologies result in mtDNA-dependent sterile inflammation associated with distinct mtDNA intracellular location. **a** NFκB target and type I IFN response gene expression in myoblasts with fragmented mitochondria (*n* = 7). **b** IL1β and IFNβ levels in cultured media from myoblasts with fragmented mitochondria (*n* = 6). **c** NFκB target and type I IFN response gene expression in myoblasts with elongated mitochondria (*n* = 7). **d** IL1β and IFNβ levels in cultured media from myoblasts with elongated mitochondria (*n* = 6). NFκB target gene expression upon mtDNA depletion in myoblasts with **e** fragmented and **f** elongated mitochondria (*n* = 6). **g** Representative immunoblot of LAMP1, TIMM23 and Tubulin in subcellular fractionations (*n* = 3). Expression of mtDNA-encoded genes relative to nuclear-encoded gene (*bActin*) in cytosolic fractions of myoblast with **h** fragmented (*n* = 7) or **i** elongated (*n* = 6) mitochondria. **a**–**d**, **i**, **h** Two-sided Students’ *t* test per gene. **e**, **f** Two-way ANOVA test and post hoc *t* tests. Data are expressed as the mean of *n* independent experiments ± SEM.*p vs. Scr <0.05. ^#^*p* vs cognate KD - ddC <0.05. **a**, **c** Created with BioRender.com. **a**–**i** Source data is provided in the Source Data File.

We studied the impact of different disturbances in mitochondrial dynamics on inflammation by generating stable knockdown (KD) C2C12 muscle cells (myoblasts) for either the fusion proteins Mfn1 and Mfn2 (Supplementary Fig. 1a, c), or the fission proteins Fis1 and Drp1 (Supplementary Fig. 1b, d), which resulted in fragmented or elongated mitochondrial networks, respectively (Supplementary Fig. 1e, f). To assess whether these alterations were linked to an inflammatory response, we analyzed the NFκB- and IFN-induced inflammatory pathways. Mfn1KD and Mfn2KD myoblasts, with fragmented mitochondrial networks, presented upregulation of NFκB target genes and unaltered expression of IFN-induced genes, except for *Ifnb* in Mfn2KD myoblasts (Fig. 1a). This was accompanied by increased secretion of IL1β secretion in both cell lines, whereas IFNβ secretion was only increased in Mfn2KD myoblasts (Fig. 1b). Further studies are required to understand the effects of Mfn2 deficiency on IFNβ secretion. Fis1- and Drp1-deficient myoblasts had elongated mitochondrial networks, and showed activation of both NFκB-dependent inflammation and the type I IFN response, with increased secretion of both IL1β and IFNβ to the cultured medium (Fig. 1d, d). To validate these observations, we modulated the mitochondrial morphology of muscle cells by different approaches: acute downregulations of mitochondrial dynamics proteins in (a) differentiated wild-type (WT) myotubes (Supplementary Fig. 2a), and (b) in WT myoblasts (Supplementary Fig. 2b); and overexpression of Drp1 or its dominant-negative form (Drp1 WT and Drp1K48A) (Supplementary Fig. 2c) in WT myoblasts. Acute downregulation of mitochondrial dynamics proteins in differentiated myotubes or myoblasts induced similar inflammatory patterns to those observed in all stable KD myoblasts assessed in this study (Supplementary Fig. 2d–g), suggesting that the inflammatory responses driven by altered mitochondrial dynamics are independent of the differentiation state of muscle cells and are not a consequence of adaptations that stable KD might undergo. Furthermore, modulation of mitochondrial morphology by overexpressing Drp1 WT or Drp1K48A led to an interesting observation in relation to its effects in inducing inflammation in myoblasts. Overexpression of Drp1 WT, leading to fragmented mitochondria via activation of fission, resulted in modest effects on the activation of inflammatory signals (Supplementary Fig. 2h). This narrowly resembles the inflammatory signature detected in Mfn1- and Mfn2KD myoblasts, where mitochondrial fragmentation is induced by inhibition of fusion. On the contrary, overexpression of Drp1K48A, leading to mitochondrial elongation via inhibition of fission, recapitulated the inflammatory phenotype observed in Fis1- and Drp1-deficient myoblast, which also show inhibition of fission (Supplementary Fig. 2h). These data indicate that modulation of mitochondrial morphology by different approaches can result in different inflammatory patterns. In fact, our data suggest that mitochondrial fragmentation induced by inhibition of fusion associates with an NFκB-dependent inflammatory response, whereas mitochondrial elongation induced by inhibition of fission results in the activation of both NFκB-dependent and the type I IFN response.

It has been shown that mtDNA acts as a mtDAMP upon mitochondrial damage, triggering inflammatory responses^6,18–22^. To assess mtDNA’s involvement in the activation of inflammation upon imbalanced mitochondrial dynamics, all stable KD myoblasts were treated with 2′,3′ dideoxycytidine (ddC), a nucleoside analog that inhibits mtDNA replication - and therefore promotes mtDNA depletion. As expected, after 72 h of ddC treatment, all stable KD myoblasts presented a remarkable depletion of mtDNA (Supplementary Fig. 3a, b). Under these conditions, control myoblasts treated with ddC did not show altered expression levels of inflammatory genes (Supplementary Fig. 3c), while the inflammatory responses observed in myoblasts with altered mitochondrial dynamics were restored (Figs. 1e, f, Supplementary Fig. 3d, e). This finding implies a role for mtDNA in activating inflammation upon altered mitochondrial dynamics. The induction of deficiency in specific mitochondrial proteins causes mislocalization of mtDNA resulting in inflammation^6,18–22^. Therefore, we hypothesized that the extra-mitochondrial location of mtDNA, upon imbalances in mitochondrial dynamics, is responsible for triggering inflammatory responses. Subcellular fractionation (Fig. 1g) and subsequent quantification of mtDNA abundance in cytosolic fractions showed that Mfn1- or Mfn2-deficient myoblasts do not show an increased presence of mtDNA sequences in the cytosol (Fig. 1h). This observation is consistent with the absence of activation of the type I IFN response in cells with fragmented mitochondrial networks. In contrast, Fis1- or Drp1-deficient myoblasts showed an enhanced abundance of mtDNA in the cytosolic fraction (Fig. 1i), which suggests that cGAS activation mediates the inflammatory response. Fluctuations of mtDNA in the cytosol were independent of the total mtDNA content in all the cell lines, except Drp1-deficient myoblasts, which showed an increased mtDNA content compared to control cells (Supplementary Fig. 4a, b). Moreover, assessment of other mitochondrial parameters, including mitochondrial membrane potential (Δψm) (Supplementary Fig. 4c, e), mitochondrial superoxide (mtROS) production (Supplementary Fig. 4d, f), oxygen consumption rates (Supplementary Fig. 4g, h) and mitochondrial mass (Supplementary Fig. 4i), did not reveal any parallelism with the inflammatory profile observed upon fragmentation or elongation of the mitochondrial network. Alterations in mitochondrial dynamics are linked to disrupted mitophagy^3,4,6,7^, and abnormal mitophagy has been in turn described to activate inflammatory responses^19,21,22^. Thus, we evaluated the mitophagic flux of stable KD myoblasts by monitoring the accumulation of mitochondrial mass upon bafilomycin A1 treatment. Strikingly, Mfn1- and Fis1KD myoblasts showed increased mitophagic flux, whereas Mfn2- and Drp1KD myoblasts exhibited unaltered mitophagic activity (Supplementary Fig. 4j, k). These results do not associate with the inflammatory profiles observed in each cell line. Therefore, these findings support the hypothesis that extra-mitochondrial mtDNA location is key in driving inflammation in these contexts. Collectively, all these data suggest that mitochondrial fragmentation and elongation are associated with distinct inflammatory profiles and that mtDNA mislocation is linked to the initiation of inflammation upon certain imbalances in mitochondrial dynamics.

### Differential DNA sensor activation underlies the inflammatory profiles upon altered mitochondrial dynamics

**Fig. 2:**
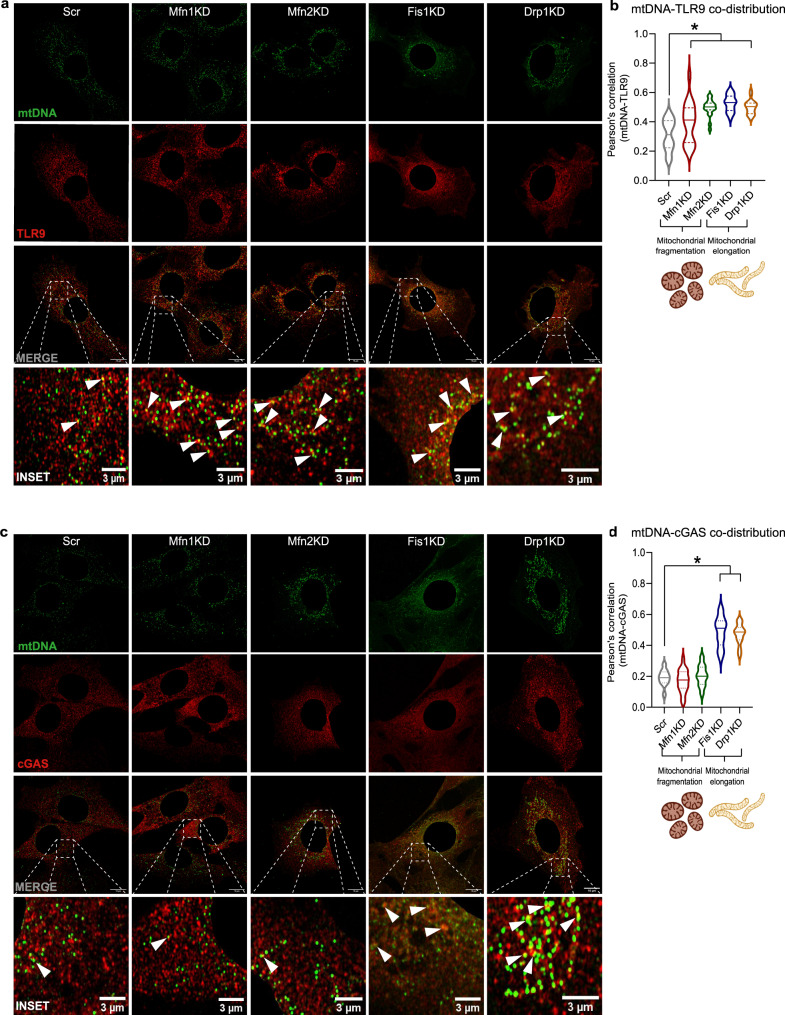
Increased co-distribution between mtDNA and DNA sensors is associated with inflammation upon altered mitochondrial dynamics. **a** Representative immunostainings of dsDNA (green) with subtraction of the nuclear signal (mtDNA), and TLR9 (red) in Scr, Mfn1KD, Mfn2KD, Fis1KD, and Drp1KD myoblasts. Scale bar 10 µm in MERGE and 3 µm in INSET. **b** Quantification of the Pearson’s correlation between mtDNA and TLR9 (*n* = 20 images per condition). **c** Representative immunostainings of dsDNA (green) with subtraction of the nuclear signal (mtDNA), and cGAS (red) in Scr, Mfn1KD, Mfn2KD, Fis1KD, and Drp1KD muscle cells. Scale bar 10 µm in MERGE and 3 µm in INSET. **d** Quantification of Pearson’s correlation between mtDNA and cGAS. (*n* = 20 images per condition). **a**, **c** White arrows point positive co-distribution. **b**, **d** Two-way ANOVA test and post-hoc *t* tests. Data are expressed as mean ± SEM. *p vs. Scr <0.05. **b**, **d** Created with BioRender.com. **b**, **d** Source data is provided in the Source Data File.

To confirm the involvement of mtDNA mislocation in the activation of intracellular inflammatory pathways upon alterations in mitochondrial morphology, we performed super-resolution confocal microscopy to detect the DNA sensors TLR9 and cGAS, together with mtDNA. The use of an anti-double-stranded DNA (dsDNA) antibody with subsequent nuclear subtraction of the signal to visualize mtDNA has been previously used^6,19,27,28^ and was further validated by assessing the co-distribution of extra-nuclear dsDNA puncta with the outer mitochondrial membrane marker TOMM20 in control myoblasts (Supplementary Fig. 5a). The co-distribution of mtDNA and TLR9 increased in all stable KD myoblasts, regardless of the resulting mitochondrial morphology (Fig. 2a, b). In contrast, the co-distribution of mtDNA and cGAS increased only in muscle cells with elongated mitochondrial networks (Fig. 2c, d). In fact, the unchanged co-distribution between mtDNA and cGAS in Mfn1KD and Mfn2KD cells validates the observation that downregulation of mitochondrial fusion protein does not lead to mtDNA leakage to the cytosol and, hence, the type I IFN response is not induced.

**Fig. 3:**
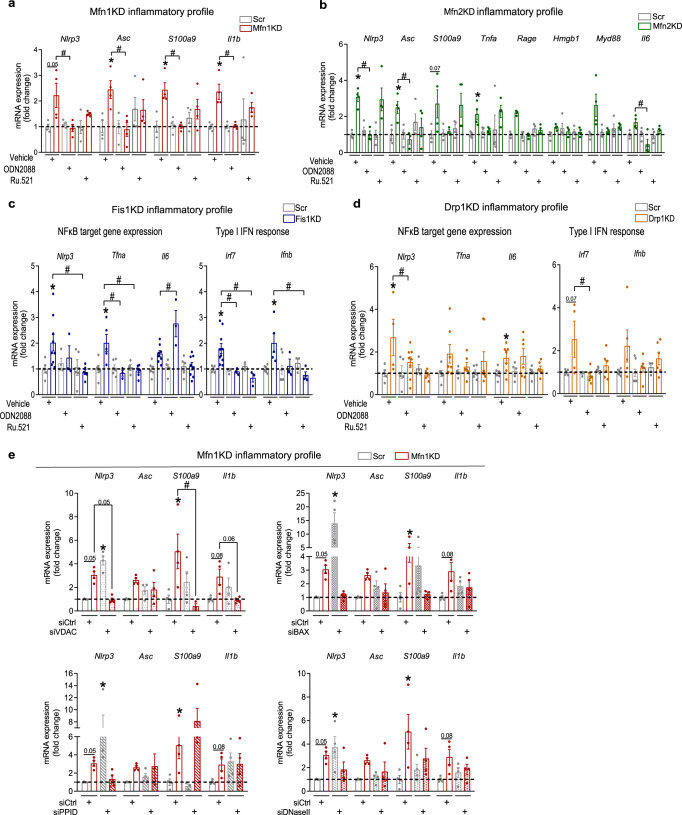
Blockade of DNA sensors rescues the inflammatory profile upon altered mitochondrial dynamics. **a** Inflammatory profile in Mfn1KD myoblasts upon ODN2088 or Ru.521 treatment (1 µM, 24 h) (*n* = 4). **b** Inflammatory profile in Mfn2KD myoblasts upon ODN2088 or Ru.521 treatment (1 µM, 24 h) (*n* = 4). **c** Inflammatory profile in Fis1KD myoblasts upon ODN2088 or Ru.521 treatment (1 µM, 24 h) (*n* = 8). **d** Inflammatory profile in Drp1KD myoblasts upon ODN2088 or Ru.521 treatment (1 µM, 24 h) (*n* = 7). **e** Inflammatory profile upon *Vdac1*, *Bax, Ppid*, or *DNaseIIa* acute downregulation in Scr and Mfn1KD myoblasts (*n* = 4). **a**–**e** Two-way ANOVA test and post-hoc *t* tests. Data are expressed as mean of *n* independent experiments ± SEM.*p vs. Scr + vehicle <0.05 and ^#^*p* vs. cognate KD + vehicle <0.05 in **a**–**d**; *p vs. Scr + siCtrl <0.05 and ^#^*p* vs. cognate KD + siCtrl <0.05 in **e**. **a**–**e** Source data is provided in the Source Data File.

To further evaluate the role of mtDNA-DNA sensor engagement upon imbalances in mitochondrial dynamics, we treated stable KD myoblasts with the antagonist of TLR9, namely ODN2088^29^, or the specific inhibitor of cGAS^30^, Ru.521. In the case of Mfn1KD and Mfn2KD myoblasts, ODN2088 treatment, and not Ru.521, normalized the mRNA levels of NFκB target genes (Fig. 3a, b), and *Ifnb* in Mfn2KD myoblasts (Supplementary Fig. 5b). Regarding Drp1- and Fis1KD myoblasts, both ODN2088 and Ru.521 blocked, to a different extent, the expression of inflammatory genes. Under conditions of Fis1 deficiency, cGAS inhibition fully restored the expression of NFκB target genes and type I IFN response genes, whereas the effects of TLR9 inhibition were moderate (Fig. 3c, Supplementary Fig. 5c). In Drp1-deficient myoblasts, both treatments marginally attenuated the expression of NFκB target genes, and type I IFN response genes (Fig. 3d, Supplementary Fig. 5d). In all, our data suggest a major involvement of cGAS in triggering inflammation upon mitochondrial elongation.

Our data indicate that the inhibition of mitochondrial fission activates the TLR9 and cGAS inflammatory pathways, and they support the notion that these pathways interact. Nonetheless, mtDNA leakage into the cytosol during the activation of inflammation upon inhibition of both mitochondrial fission proteins appears to be a key factor. In fact, recent evidence on the mechanisms governing mtDNA leakage into the cytosol upon mitochondrial stress suggests that potentiated transport of pyrimidines inside mitochondria promotes mtDNA accumulation, which induces the escape of mtDNA sequences to the cytosol^18^. To determine whether the transport of pyrimidines was responsible for the mtDNA leakage in Fis1- and Drp1-deficient myoblasts, we measured the expression of the pyrimidine carrier SLC25A33, and YME1L, the protease responsible for SLC25A33′s processing. In keeping with recent observations^18^, YME1L-deficient myoblasts showed an enhanced expression of SLC25A33 (Supplementary Fig. 6a). However, Drp1-deficient myoblasts did not present changes in the expression levels of either YME1L or SLC225A33; whereas, the downregulation of Fis1 promoted an increase in SLC25A33 (Supplementary Fig. 6a). Hence, the mechanisms promoting mtDNA exit from mitochondria in Fis1-deficient myoblasts could be driven by pyrimidine imbalance via SLC25A33. Nevertheless, Fis1-deficient myoblasts exhibit unchanged total mtDNA abundance, in contrast to YME1LKD myoblasts (Supplementary Fig. 6b), suggesting some differences in the adaptive responses induced by these manipulations

To further investigate the mechanisms responsible for mtDNA mislocation upon imbalanced mitochondrial dynamics, we analyzed the implication of mitochondrial pores, such as VDAC^31^, Bax/Bak^32–34^ and the mitochondrial permeability transition pore (mPTP)^35^, whose involvement in mtDNA mislocation in the cytosol has been previously reported. To this aim, we performed acute downregulation of the key pore components—i.e., VDAC1 (siVDAC), Bax (siBAX), and cyclophilin D (siPPID) (Supplementary Fig. 6c–e)—in Mfn1KD myoblasts, as a model for mitochondrial fragmentation and endosomal mtDNA location, and Fis1KD myoblasts, as a model for mitochondrial elongation and cytosolic mtDNA. Our data show that NFκB target gene expression was rescued upon downregulation of VDAC1 in Mfn1KD myoblasts (Fig. 3e). Bax downregulation lead to a certain degree of rescue effect, whereas cyclophilin D downregulation did not (Fig. 3e). Thus, our data suggest that VDAC pores are likely to be involved in the processes driving inflammation in Mfn1KD myoblasts. In the case of the Fis1 deficiency, the downregulation of VDAC1, Bax, or PPID did not promote the rescue of either NFκB target genes or the type I IFN response genes. If anything, a surprising aggravation of the observed inflammatory phenotype was detected in double-KDs (Supplementary Fig. 6f). These data indicate that the mechanisms by which mtDNA leaks to the cytosol in Fis1KD myoblasts are more complex than what has been reported so far in other similar models of cytosolic mtDNA^18,20,31,32,34,35^. We propose that such mechanisms might involve the action of all kinds of pores in a synergistic manner, which represents an unprecedented scenario requiring much effort to comprehend.

It has been reported that engagement of TLR9 by recognition of DNA sequences to downstream inflammatory signals depends on the action of endosomal DNase II^36^. With aim of providing further mechanistic insights, regarding the link between mitochondrial fragmentation and the triggering of TLR9, we assessed the implication of DNase II under conditions of Mfn1 deficiency. Hence, we quantified the expression levels of NFκB target genes upon deficiency of DNase II (*DNaseIIa*) in Mfn1KD (Supplementary Fig. 6g). According to our data, a mild tendency towards rescue of the expression levels of NFκB target genes could be detected in conditions of Mfn1 deficiency (Fig. 3f). These results could be indicative of the implication of DNase II in driving inflammation in Mfn1KD-induced mitochondrial fragmentation.

Taken together, our data demonstrate that opposite mitochondrial morphologies are associated with the activation of distinct intracellular inflammatory pathways. The mislocation of mtDNA appears as a hub in the induction of inflammation (Supplementary Fig. 6h). On one hand, mitochondrial fragmentation mediated by downregulated Mfn1 or Mfn2 is linked to endosomal mtDNA location likely mediated by VDAC pores, TLR9 activation, and NFκB-mediated inflammation. On the other hand, myoblasts with elongated mitochondrial networks due to Drp1 or Fis1 downregulation, show a more complex scenario. Both TLR9 and cGAS contribute, to different extents, to trigger inflammation in Fis1- and in Drp1-deficient myoblasts, and under these conditions, mtDNA leakage to the cytosol, probably by different mechanisms, appears as a key factor.

### Mitochondria establish contacts with early endosomes upon Mfn1 downregulation

**Fig. 4:**
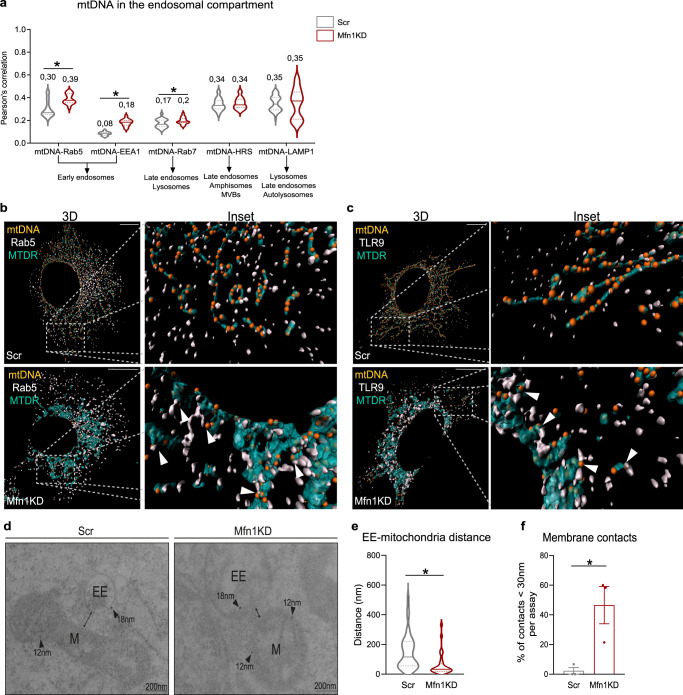
Mfn1 deficiency causes preferential location of mtDNA and mitochondria in early endosomes and promotes close contacts between Rab5^+^ early endosomes and mitochondria. **a** Quantification of Pearson’s correlation between mtDNA and endosomal markers (*n* = 20 images per condition, except for mtDNA-LAMP1, where *n* = 39). Representative 3D reconstructions of immunostainings using dsDNA with nuclear subtraction (mtDNA, orange), mitochondria with Mitotracker Deep Red (turquoise) and **b** Rab5 (white) or **c** TLR9 (white) (Scale bar,10 µm). **d** Representative images of immunogold staining of Rab5 (gold particle 18 nm) and SdhA (gold particle 12 nm) in Scr- and Mfn1KD myoblasts (Scale bar, 200 nm). **e** Quantification of the distance between marked early endosomes and mitochondria in Scr (*n* = 38 contacts) and Mfn1KD myoblasts (*n* = 31 contacts). **f** Percentage of measured contacts <30 nm in Scr- and Mfn1-deficient muscle cells (*n* = 3, each point represents the mean of quantifications obtained in three independent experiments). **b**, **c** Arrows point to positive co-distribution. **d** Arrows point gold particles. **a**, **e**, **f** Two-sided Students’ *t* test. Data are expressed as the mean of *n* independent experiments ± SEM.*p vs. Scr <0.05. **a**, **e**, **f** Source data is provided in the Source Data File.

Since Mfn1 or Mfn2 deficiency triggers a similar inflammatory response characterized by the activation of NFκB—and not the type I IFN response—in a TLR9-dependent manner, we focused on describing the molecular mechanisms involved. Given that some of the cellular functions of Mfn2 go beyond mitochondrial fusion^37^, we further explored Mfn1-deficient myoblasts to elucidate the molecular mechanisms involved. Thus, to characterize the type of endosome in which mtDNA is more likely to encounter TLR9 in Mfn1-deficient myoblasts, we performed a battery of co-distribution studies between mtDNA and several endosomal markers, including Rab5 and EEA1 for early-endosomes, Rab7 for late endosomes, HRS for intermediate species, and LAMP1 for late endosomes, lysosomes and autolysosomes. Strikingly, increased co-distribution between mtDNA and early endosomal markers, i.e., Rab5 and EEA1, was detected in Mfn1KD myoblasts, compared to control myoblasts (Fig. 4a, Supplementary Fig. 7a).

To approach how mtDNA interacts with early endosomes, we performed triple staining assays of mtDNA, mitochondria (Mitotracker Deep Red, MTDR), and Rab5 (Supplementary Fig. 7b) or TLR9 (Supplementary Fig. 7c) in control and Mfn1-deficient myoblasts. 3D reconstructions of these images revealed that mtDNA contacts with either Rab5 (Fig. 4b) or TLR9 (Fig. 4c) occur in the presence of mitochondria. Increased co-distribution between MTDR and TLR9 or Rab5 in Mfn1-deficient myoblasts confirmed these results (Supplementary Fig. 7d). These data suggest either a possible engulfment of mitochondria by Rab5^+^ vesicles^38^, the inclusion of Rab5^+^ vesicles in mitochondria^39^, or the transfer of mtDNA sequences through mitochondria-early endosome contacts^40^. To identify the possible interactions between mitochondria and early endosomes, we performed immunogold assays in control and Mfn1-deficient myoblasts. To this end, we tagged mitochondria with SdhA (12 nm) and early endosomes with Rab5 (18 nm). These assays showed that physical approximation and close contacts between Rab5^+^ endosomes and mitochondria are more likely to occur in Mfn1-deficient myoblasts (Fig. 4d). Moreover, quantification of the distance between Rab5^+^ endosomes and mitochondria revealed a mean distance of 150 nm in control cells and 63 nm in Mfn1KD myoblasts (Fig. 4e), with 2 and 47% of the measured contacts presenting a distance <30 nm, respectively, (Fig. 4f). These findings indicate that Mfn1-deficient myoblasts show more mitochondria-early endosome contact sites compared to control cells. Moreover, based on our data showing that Mfn1KD-dependent inflammatory signature was reduced after VDAC downregulation, we propose that VDAC pores are implicated in the transfer of mtDNA through mitochondria-early endosome contact sites.

### Rab5C binds to Mitofusins driving early endosome-mitochondria contacts

**Fig. 5:**
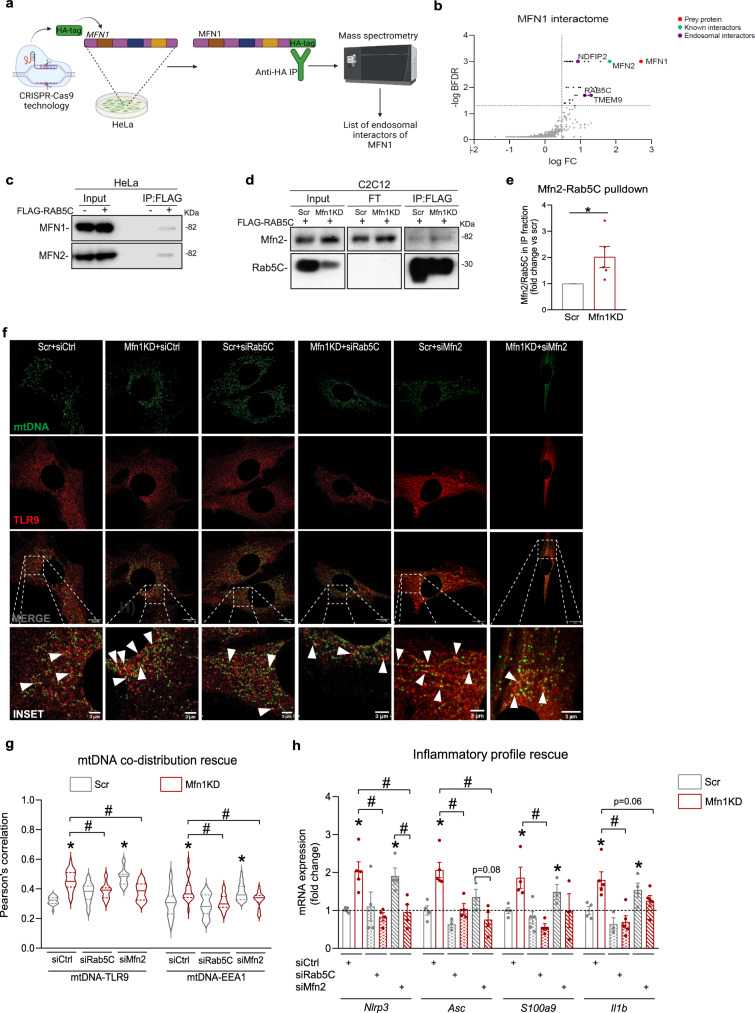
Interaction between Mfn2 and Rab5C promotes mitochondria-early endosomal contacts upon Mfn1 deficiency, driving mtDNA and TLR9-dependent NFκB-mediated inflammation. **a** Workflow of the generation of the MFN1-HA HeLa cell line, HA immunoprecipitation, and mass spectrometry analysis. **b** Graphical representation of MFN1 interacting candidates organized according to their fold change (FC) and Bayesian False Discovery Rate (BFDR). Identified in purple are the endosomal interactors of MFN1, and in green a known MFN1 interactor. **c** MFN1 and MFN2 representative immunoblots of immunoprecipitation (IP) in WT HeLa cells overexpressing FLAG-RAB5C (*n* = 6). **d** Mfn2 and Rab5C representative immunoblots of input, flowthrough (FT), and eluate (IP) fractions of FLAG immunoprecipitation in Scr and Mfn1KD muscle cells overexpressing FLAG-RAB5C (*n* = 5). **e** Quantification of the Mfn2/Rab5C ratio in the IP fraction (*n* = 5). (Representative immunostainings of dsDNA (green) with subtraction of the nuclear signal (mtDNA), and TLR9 (red) (Scale bar 10 µm in MERGE and 3 µm in INSET) upon siCtrl, siRab5C or siMfn2 transfection in Scr and Mfn1KD cells. White arrows point positive co-distribution. **g** Quantification of Pearson’s correlation between mtDNA and TLR9 or EEA1 upon siCtrl, siRab5C, or siMfn2 transfection in Scr and Mfn1KD cells (*n* = 20 images per condition). **h** NFκB target gene expression upon siCtrl, siRab5C, or siMfn2 transfection in Scr and Mfn1KD cells (*n* = 5). **e** Two-sided Students’ *T* test, **g**, **h** Two-way ANOVA test and post-hoc *t* tests. Data are expressed as mean of *n* independent experiments ± SEM.*p vs. Scr <0.05 in **e**; *p vs. Scr + siCtrl <0.05 and ^#^*p* vs. Mfn1KD + siCtrl <0.05 in **g**, **h**. **a** Created with BioRender.com. **c**–**e**, **g**, **h** Source data is provided in the Source Data File.

Mitochondria-endosome contacts and their protein interactors are involved in several cellular processes, including iron uptake and transfer to mitochondria^40^, melanogenesis^41^, and the fusion of lysosomes to autophagosomes^42^. However, the interactors between mitochondria and early endosomes, and their impact on the activation of intracellular inflammatory pathways remained unexplored. To address this gap, we performed immunoprecipitation of endogenously expressed MFN1 followed by mass spectrometry analysis (IP-MS) to obtain its interactome. To this end, we used CRISPR-Cas9 technology to allow the incorporation of an HA-tag sequence in *MFN1* alleles of HeLa cells (Fig. 5a, Supplementary Fig. 8a). Insertion of an HA-tag in *MFN1* alleles allowed pulldown against HA in conditions of endogenous levels of MFN1 (Fig. 5a, Supplementary Fig. 8b). Among the partners of MFN1 identified by IP-MS (PRIDE dataset identifier PXD037935), three candidate partners localized in the endosomal compartment (Fig. 5b), of which RAB5C was the only early endosomal marker. To validate this interaction, FLAG-RAB5C was overexpressed and immunoprecipitated in WT HeLa cells (Supplementary Fig. 8c). We observed that MFN1 co-immunoprecipitated with RAB5C in these conditions, and, strikingly, MFN2 was also found in the immunoprecipitated fraction (Fig. 5c). These results allowed us to hypothesize that upon mitochondrial fragmentation, the interaction between MFN2/MFN1 and RAB5C enhances mitochondria-endosome contacts, which facilitates the approximation of mtDNA to TLR9. To validate these observations in Mfn1KD myoblasts, we first quantified protein levels of Mfn2 and Rab5C in basal conditions. Interestingly, Mfn1 deficiency in C2C12 myoblasts led to the increased total content of Mfn2 and Rab5C proteins (Supplementary Fig. 8d), which could potentially favor their interaction. As expected, FLAG-RAB5C overexpression followed by FLAG immunoprecipitation confirmed that Mfn2-Rab5C interact in control myoblasts (Fig. 5d). Strikingly, Mfn1-deficient myoblasts showed an increased immunoprecipitation of Mfn2 together with Rab5C (Fig. 5d, e), suggesting an enhanced interaction of these two proteins in conditions of Mfn1 deficiency. This strengthened interaction might be responsible for a closer approximation between early endosomes and mitochondria in Mfn1 deficiency-induced mitochondrial fragmentation.

To assess the biological effect of this interaction on triggering inflammation in Mfn1KD myoblasts, we examined the location of mtDNA and NFκB target gene expression upon acute downregulation of Rab5C or Mfn2 (Supplementary Fig. 8e). Surprisingly, reduced levels of either Rab5C or Mfn2 in Mfn1KD myoblasts reduced the co-distribution between mtDNA and TLR9 (Fig. 5f, g), or between mtDNA and the early endosomal marker EEA1 (Fig. 5g, Supplementary Fig. 8f) which resulted in normalized NFκB target gene expression (Fig. 5h). Therefore, these observations confirm that upon mitochondrial fragmentation, mitochondria-early endosome contacts, mediated by interactions between Rab5C and mitofusins, are required for mtDNA encounter with TLR9, and the activation of NFκB-mediated inflammation.

In all, our data describe a biological process linking mitochondrial fragmentation induced by Mfn1 repression and NFκB-mediated inflammation. This process relies on the interaction between Mfn2/Mfn1 and Rab5C, which results in more mitochondria-early endosome contacts where VDAC1 potentially plays a role. This interaction acts as a prerequisite to mtDNA-TLR9 engagement, leading to NFκB-dependent inflammation (Supplementary Fig. 8g).

### Mfn1 links inflammation to muscle atrophy and compromised physical performance in vivo

**Fig. 6:**
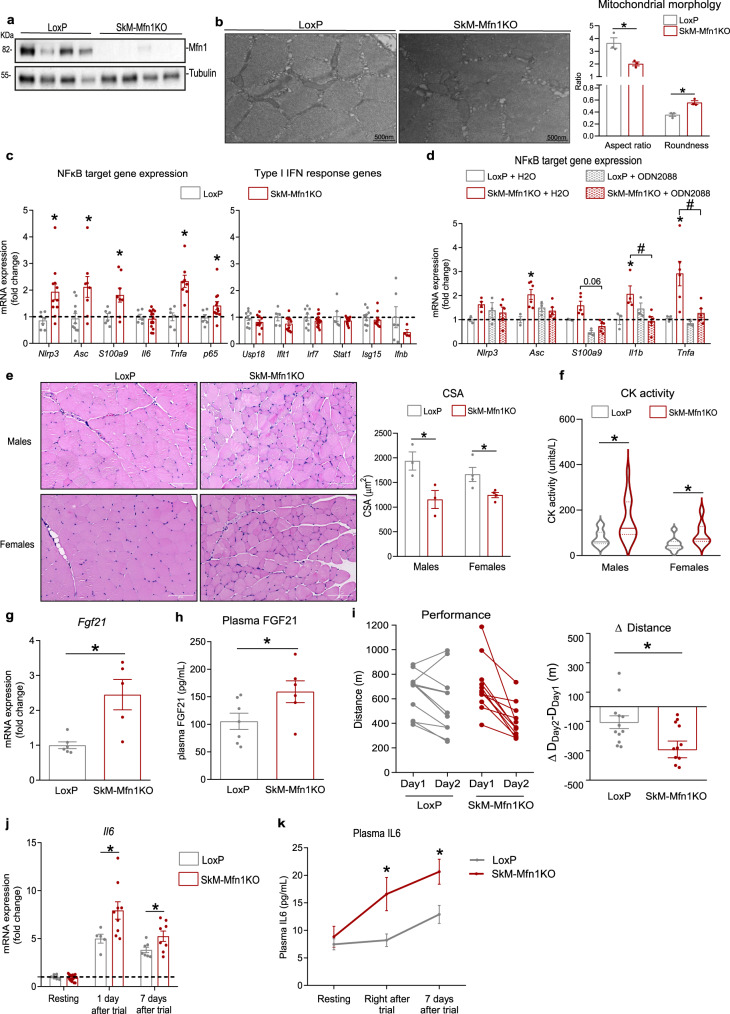
Specific ablation of Mfn1 in skeletal muscle promotes TLR9-dependent NFκB-dependent inflammation, muscle atrophy, reduced physical performance, and enhanced IL6 response to exercise. **a** Mfn1 and Tubulin representative immunoblot in total homogenates of quadriceps muscles of LoxP (*n* = 4 mice) and SkM-Mfn1KO (*n* = 4 mice) male and female mice. **b** TEM images from cross-sectional sections of quadriceps muscles of LoxP and SkM-Mfn1KO male mice (*n* = 4 mice) and quantification of parameters representing mitochondrial morphology (Scale bar, 500 nm). **c** NFκB target and type I IFN response gene expression in quadriceps muscles of LoxP (*n* = 8 mice) and SkM-Mfn1KO mice (*n* = 14 mice). **d** NFκB target gene expression in quadriceps muscles of endotoxin-free water- or ODN2088-treated (100 µg/mice, necropsy 48 h after injection) LoxP (*n* = 4 mice) or SkM-Mfn1KO male mice (*n* = 6 mice). **e** Representative images of hematoxylin/eosin staining of cross-sectional sections of gastrocnemius muscles (Scale bar, 100 µm) and quantification of the CSA (*n* = 3 mice; 4 areas per mice; 30 fibers per area). **f** CK activity in plasma samples (*n* = 16 mice). **g** *Fgf21* mRNA levels in quadriceps muscles of LoxP (*n* = 6 mice) and SkM-Mfn1KO male mice (*n* = 5 mice). **h** Plasma FGF21 levels in LoxP (*n* = 7 mice) and SkM-Mfn1KO male mice (*n* = 6 mice). **i** Distance run on the treadmill test and the difference in the distance run between day 2 and day 1 in LoxP (*n* = 12 mice) and SkM-Mfn1KO male mice (*n* = 11 mice). **j** *Il6* expression levels in quadriceps muscles and **k** plasma IL6 levels of LoxP and SkM-Mfn1KO male mice at resting conditions or at different time-points after the treadmill test (*n* = 14 mice). **b**, **e**–**k** Two-sided Students’ *T* test, **c** Two-sided Students’ T-test per gene, and **d** two-way ANOVA test and post hoc *t* tests. Data are expressed as mean ± SEM. *p vs. Lox*P* <0.05. **a**–**k** Source data is provided in the Source Data File.

To evaluate the physiological implications of Mfn1 downregulation in muscle health, we used. tamoxifen-inducible HSA-Cre driven skeletal muscle-specific Mfn1 knockout (SkM-Mfn1KO) mice. At 12 weeks of age, mice were fed with tamoxifen-enriched diet for 2 weeks, after which tamoxifen washout was allowed by provision of chow diet for 2 weeks. This method proved efficient to ablate Mfn1 exclusively in skeletal muscle only in the Cre-positive mice treated with tamoxifen (Fig. 6a, Supplementary Fig. 9a, b). As a result of Mfn1 ablation in skeletal muscles, fragmented mitochondria were detected by transmission electron microscopy in quadriceps muscles of SkM-Mfn1KO mice, shown by a reduction in the aspect ratio and increase roundness of mitochondria (Fig. 6b). Body weight, food intake, and fat and lean mass were not altered in male or female (Supplementary Fig. 9c–e) mice after 2 months of inducing Mfn1 depletion.

Under these conditions, the induction of inflammation was assessed in quadriceps muscles. Ablation of Mfn1 in skeletal muscles led to NFκB-mediated inflammation especially in males (Fig. 6c), with limited effects in females (Supplementary Fig. 9f). Furthermore, Mfn1-depleted muscles did not show induced expression of type I IFN response genes in either sex (Fig. 6c and Supplementary Fig. 9f) and the inflammatory response was rescued upon a single injection of the TLR9 antagonist ODN2088 in males (Fig. 6d). These results indicate that Mfn1 ablation in vivo results in TLR9-driven NFκB-mediated inflammation, in keeping with our results in Mfn1-deficient muscle cells. Surprisingly, negative staining of the macrophage marker F4/80 and IL1β by immunohistochemistry assays in gastrocnemius sections (Supplementary Fig. 9g), along with unchanged plasma levels of the pro-inflammatory cytokines IL6 and TNFα (Supplementary Fig. 9h), revealed that the inflammation in SkM-Mfn1KO mice was localized within the muscle fibers. To evaluate muscle health in these animals, we studied the cross-sectional area (CSA) of gastrocnemius muscles using hematoxylin/eosin staining. Quantification of the CSA showed a remarkable 50% and 30% decrease in SkM-Mfn1KO males and females, respectively (Fig. 6e), compared to their LoxP littermates. Corroborating these data, we detected increased plasma activity of the muscle damage marker creatine kinase (CK) in both sexes (Fig. 6f). Furthermore, muscle gene expression (Fig. 6g) and plasma protein levels (Fig. 6h) of a muscle stress sensor, the myokine FGF21, were increased in males despite the expression of atrophy-related genes (atrogenes) remaining unchanged (Supplementary Fig. 9i). Taken together, skeletal muscle Mfn1 ablation triggers muscle inflammation and muscle atrophy, thereby suggesting that the SkM-Mfn1KO mouse is a model of inflammatory myopathy.

To assess potential physiological outcomes of inflammation and muscle atrophy in SkM-Mfn1KO mice, we evaluated their physical performance by subjecting them to two consecutive rounds of exercise on a treadmill until exhaustion. Although SkM-Mfn1KO and their LoxP littermates did not present differences in physical performance on day 1 of the trial, SkM-Mfn1KO mice showed a decrease in the distance run on day 2 (Fig. 6i). These data suggest that the tissue repair mechanisms triggered upon exercise-induced damage are potentially impaired in SkM-Mfn1KO mice, which may have prevented them from performing like their LoxP littermates on day 2. To further address this notion, we focused on IL6, since this cytokine is physiologically produced and secreted by skeletal muscle upon exercise^43,44^. To this end, we studied whether the inflammation present in the muscles of SkM-Mfn1KO mice in resting conditions exacerbated the IL6 response to exercise, resulting in an enhanced systemic inflammatory response. Quantification of mRNA levels in quadriceps (Fig. 6j) and plasma levels (Fig. 6k) of IL6 one day and seven days after the end of the trial revealed increased IL6 response upon exercise in SkM-Mfn1KO, which could be a major driver of the impairment of tissue repair mechanisms and reduced physical performance in this animal model.

**Fig. 7:**
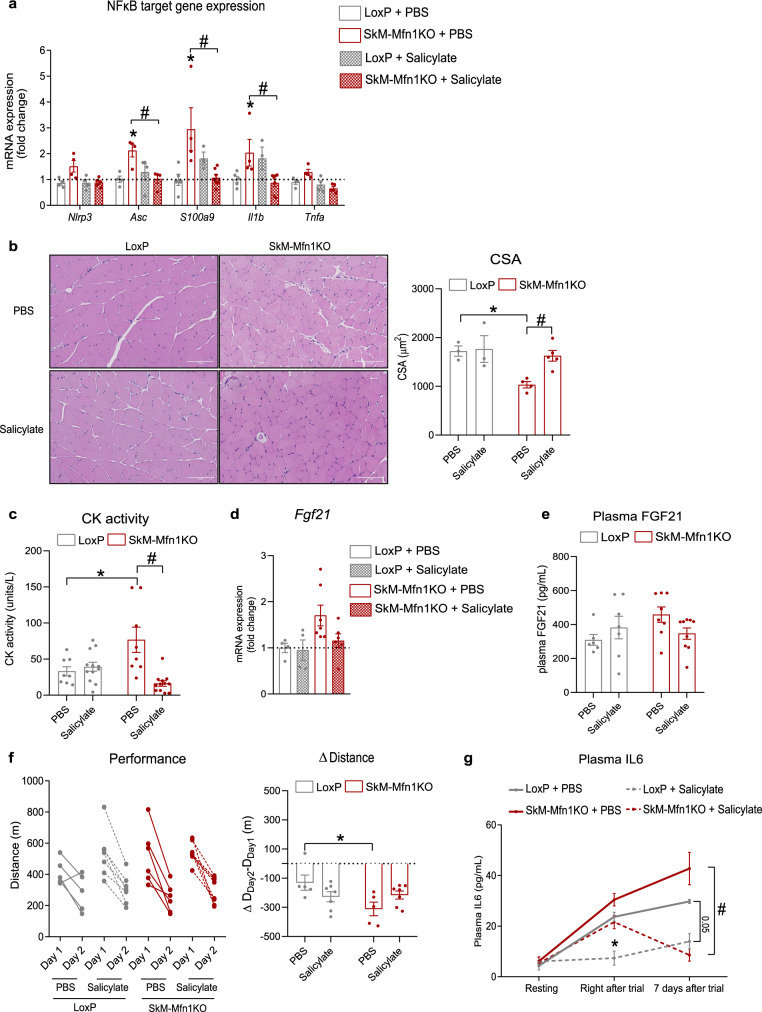
Chronic anti-inflammatory treatment normalizes muscle inflammation, rescues muscle atrophy, ameliorates physical performance, and improves exercise-induced IL6 response. **a** NFκB target gene expression in quadriceps muscles of PBS- or salicylate-treated (200 mg/kg, 28 days) LoxP (*n* = 4 mice) and SkM-Mfn1KO male mice (*n* = 5 mice). **b** Representative images of hematoxylin/eosin staining of cross-sectional sections of gastrocnemius muscles (Scale bar, 100 µm) and quantification of the CSA from PBS- or salicylate-treated mice (*n* = 3 mice; 4 areas per mice; 30 fibers per area). **c** CK activity in plasma samples in LoxP (*n* = 12 mice) and SkM-Mfn1KO PBS- or salicylate-treated male mice (*n* = 12 mice). **d** *Fgf21* mRNA levels in quadriceps muscles of LoxP (n = 5 mice) and SkM-Mfn1KO PBS- or salicylate-treated male mice (*n* = 7 mice). **e** Plasma FGF21 levels in LoxP (*n* = 7 mice) and SkM-Mfn1KO PBS- or salicylate-treated male mice (*n* = 9 mice). **f** Distance run on the treadmill test and the difference in the distance run between day 2 and day 1 in LoxP (*n* = 7 mice) and SkM-Mfn1KO PBS- or salicylate-treated male mice (*n* = 7 mice). **g** Plasma IL6 levels of LoxP and SkM-Mfn1KO PBS- or salicylate-treated male mice at resting conditions or at different time-points after the treadmill test (*n* = 8 mice). **a**–**g** Two-way ANOVA test and post hoc *t* tests. Data are expressed as mean ± SEM. *p vs. Lox*P* + PBS < 0.05, ^#^*p* vs. SkM-Mfn1KO + PBS < 0.05. **a**–**g** Source data is provided in the Source Data File.

Finally, to identify the contribution of TLR9-triggered NFκB-mediated inflammation upon Mfn1 ablation in skeletal muscles to the development of muscle atrophy and compromised physical performance, we treated mice for 28 days with sodium salicylate (200 mg/kg per day), anti-inflammatory agent with inhibitory activity on NFκB^45^. Salicylate treatment normalized the expression of NFκB target genes in SkM-Mfn1KO mice (Fig. 7a), thereby validating the anti-inflammatory effect of sodium salicylate. Importantly, chronic anti-inflammatory treatment also normalized the CSA (Fig. 7b), as well as the plasma CK activity (Fig. 7c) in SkM-Mfn1KO mice. Furthermore, salicylate treatment rescued the muscle mRNA levels and plasma levels of FGF21, although this rescue did not reach statistical significance (Fig. 7d, e). Assessment of the impact of the anti-inflammatory treatment upon exhaustive exercise on a treadmill revealed improved physical performance in SkM-Mfn1KO mice (Fig. 7f). To evaluate whether this improved performance was accompanied by a normalized exercise-induced systemic inflammatory response mediated by IL6, we quantified plasma levels of this cytokine. Although both salicylate-treated and PBS-treated SkM-Mfn1KO mice exhibited increased plasma IL6 one day after the end of the trial, plasma IL6 levels were normalized seven days after in salicylate-treated SkM-Mfn1KO (Fig. 7g). These data suggest that pre-existing low-grade muscle inflammation compromises muscle homeostasis and fitness enabling an exacerbated IL6 response to exercise, which may also compromise muscle repair upon exercise-induced damage.

## Discussion

Mitochondrial dynamics regulate mitochondrial homeostasis through the modulation of multiple elements such as organelle interaction and mitochondrial morphology. In this study, we provide evidence that mitochondrial dynamics also controls the activation of intracellular inflammatory pathways. Our conclusion is based on a number of observations, namely that: a) repression of the mitochondrial fusion proteins Mfn1 or Mfn2 induces mitochondrial fragmentation and TLR9-dependent NFκB activation; and b) Drp1 or Fis1 repression causes mitochondrial elongation and both NFκB-dependent and type I IFN inflammatory responses. Mitochondrial elongation was characterized by mislocalized mtDNA in the cytosol and activation of the DNA sensors TLR9 or cGAS. In all, our data show that opposite imbalances in mitochondrial morphology lead to clearly different inflammatory responses both in myoblasts and in myotubes (Supplementary Fig. 6h).

Under conditions of mitochondrial elongation induced by Fis1- or Drp1 deficiency in myoblasts, we document both the activation of NFκB and also a type I IFN response, which was accompanied by mtDNA leakage to the cytosol. Increasing evidence supports the link between mitochondrial dysfunction and the induction of sterile inflammatory responses mediated by cytosolic mtDNA in non-immune cells. For instance, downregulation of the mitophagic proteins FUNDC1 in liver^21^, PINK1 or IRGM1 in whole-body KO models^23^, and TFAM in MEFs^20^ have been reported to induce similar inflammatory responses. In fact, TFAM-deficient MEFs with mitochondrial hyperfusion, also showed mtDNA leakage to the cytosol and cGAS activation, which goes in parallel with the phenotype observed in Drp1KD and Fis1KD cells. However, in contrast with Drp1KD and Fis1KD cells, TFAM ablation did not induce TLR9-dependent inflammatory signals, which may be due to cell-specific mechanisms for DNA sensors activation. Interestingly, under these conditions, Mfn1 depletion rescued cGAS activation, which suggested a possible relationship between mitochondrial morphology and mtDNA mislocation. More recently, we have reported that depletion of the mitophagy protein BNIP3 in myotubes results in mitochondrial fragmentation and an NFκB-dependent inflammation^19^, triggered by the accumulation of defective mitochondria in late endosomes and leading to the encounter of mtDNA and TLR9. These data further prove our hypothesis that mitochondrial dynamics play a role in mitigating sterile inflammatory responses in muscle.

Nevertheless, at this point, the specific molecular mechanisms linking mtDNA mislocation and inflammation remained unknown. Although it is clear that inhibition of mitophagy or mtDNA instability triggers the release of mtDNA to the cytosol, ultimately leading to inflammation, the involvement of mitochondrial dynamics in mtDNA mislocation had not been addressed. In our study, mtDNA release to the cytosol was detected in cells with elongated mitochondria as a result of Fis1 or Drp1 deficiency, thus providing new insights into the variety of causes underlying mtDNA release. Furthermore, the involvement of mitochondrial pores—i.e. VDAC, Bax/Bak and the mPTP—in the release of mtDNA to the cytosol has been well reported in different models^18,31–35^. However, here we describe that under conditions of Fis1 deficiency-induced mtDNA leakage, none of the mentioned pores has a key role in allowing the exit of mtDNA, allowing us to hypothezise that, in this scenario, a more complex mechanism is taking place, perhaps involving the synergic participation of different. Besides, we detected some differences in the processes activated in response to Fis1- or Drp1 deficiency. It has recently been reported that downregulation of the mitochondrial protease YME1L causes the upregulation of the pyrimidine transporter SLC25A33, leading to an increased pyrimidine pool and resulting in mtDNA escape, which activates cGAS^18^. In this regard, we found that Fis1 deficiency caused a pattern of changes similar to those reported for YME1L. In contrast, Drp1 deficiency did not enhance the expression of SLC25A33 but increased total mtDNA abundance. Furthermore, we noted differences in the rescue effects on the inflammatory profile upon inhibition of cGAS or TLR9 in Fis1KD and in Drp1KD myoblasts. Given these data, we support the notion that Fis1 or Drp1 repression leads to inflammation through the activation of distinct mechanisms, one of them SLC25A33-dependent, which ultimately results in the stimulation of cGAS and TLR9.

Here we have also uncovered key processes by which mitochondrial fragmentation induces TLR9 activation. In fact, we report that Mfn1 or Mfn2 downregulation leads to the endosomal location of mtDNA, allowing its recognition by TLR9, which results in an NFκB-dependent inflammatory response. Importantly, we previously reported that Opa1 knockdown in C2C12 myoblasts^6^ and BNIP3 deficiency in myotubes^19^ also results mitochondrial fragmentation and NFκB-driven inflammation, mediated by mtDNA recognition by TLR9. This aligns with our observations regarding Mfn1 or Mfn2 deficiency, allowing us to formulate a general model for mitochondrial fragmentation-induced inflammation, characterized by the involvement of mtDNA-TLR9 engagement. In order to delineate the molecular mechanisms of such model, in the present work, we chose to study under conditions of Mfn1 deficiency, since it has only one reported functional activity—+i.e., mitochondrial fusion—whereas Mfn2 and Opa1 have functions beyond this process. To study the molecular events operating upon Mfn1 deficiency-induced mitochondrial fragmentation, we screened for mtDNA co-distribution with markers from distinct endosomal vesicles. Our data show that early endosomal markers such as Rab5 and EEA1 exhibit an enhanced co-distribution with mtDNA and mitochondria upon Mfn1 deficiency. In addition, immunogold analysis confirmed an enhanced abundance of mitochondria-early endosomes contact sites. This is an interesting observation since various processes are linked to the interaction between endosomes and mitochondria. For instance, “kiss and run” interactions between these two organelles have been reported to facilitate direct iron transfer in erythroid cells^40^. Close contacts between late endosomes or lysosomes and mitochondria have been reported in response to hypoxia in adenocarcinoma cells^46^, as well as melanosome-mitochondria contacts during melanosome formation^41^. Furthermore, late endosomal targeting of mitochondria is also a common feature during apoptosis signaling^47^. Also, several endosomal-dependent mitophagy pathways have recently been described, including studies involving vesicles positive for Rab5^38^, Rab7^48^, Rab11A^49^, or CARP2^50^. More recently, it has been reported that a component of the retromer responsible to transport select cargo between different endosomes, VPS35, is involved in an mtDNA-selective degradation process in conditions where mtDNA holds mutations^51^.

Immunoprecipitation of endogenous Mfn1 followed by mass spectrometry also revealed that the endosomal protein Rab5C is a common partner of Mfn1 and Mfn2 proteins, and that dampening Rab5C-Mfn2 interactions reduces mtDNA co-distribution with TLR9 and EEA1, and normalizes the resulting NFκB-mediated inflammation in Mfn1KD myoblasts. Although contacts between mitochondria and other organelles, including late endosomes, have been reported to be mediated by Mfn2^40–42^, the relevance of these interactions in triggering stress responses remained unexplored. In this regard, our data not only describe an early endosomal partner for Mfn2, but also highlight the importance of these interactions in triggering inflammatory responses upon mitochondrial fragmentation. Here, we document that the interaction between mitochondria and the endosomal compartment drives the inflammatory outcome in Mfn1KD myoblasts, as previously shown in Opa1KD myoblasts^6^, and we speculate the same in conditions of Mfn2 deficiency. In conclusion, we propose that the close contact between mitochondria and early endosomes is a requirement for the approximation of mtDNA to TLR9, engaging the NFκB-mediated inflammation in conditions of mitochondrial fragmentation induced by the deficiency of either mitochondrial fusion proteins (Supplementary Fig. 8g). Furthermore, our data show that NFκB target gene expression was rescued upon downregulation of VDAC1 in Mfn1KD myoblasts. Bax downregulation lead to a certain degree of rescue effect, whereas PPID downregulation did not exert any. Therefore, our data suggest that VDAC1 is likely to be involved in the processes driving inflammation in Mfn1KD myoblasts. Interestingly, VDAC pores have been reported to locate in the juxtaposition of the interactions between mitochondria and different endosomal species upon certain conditions^46^, thus inferring a possible role for VDAC in mitochondria-endosome contacts. Hence, our findings would provide further evidence for this hypothesis.

Given the role of mitochondrial dynamics in regulating mitochondrial function and mitophagy, it is conceivable that alterations in these processes could be involved in triggering inflammation upon mitochondrial dynamics disturbances. Here, we show that indeed, the different manipulations induced by repressing Mfn1, Mfn2, Drp1, or Fis1 lead to very different patterns of alterations in mitochondrial membrane potential, mitochondrial superoxide production, mitochondrial mass, mitochondrial respiration, or mitophagy, which does not explain the inflammatory response observed in each condition. In contrast, we find that the inflammatory responses depend on the presence of mtDNA, which suggests that those changes in mitochondrial function and quality are consequences of adaptations in mitochondrial biology that are not directly related to the inflammatory response.

As discussed above, mitochondrial stress can trigger sterile inflammation by inducing mtDNA mislocation and allowing mtDNA recognition by DNA sensors, mitochondrial dynamics are essential in maintaining mitochondrial homeostasis, and muscle inflammation and atrophy are hallmarks of impaired muscle health^52^. However, the molecular connections between mitochondrial dynamics and mtDNA-dependent inflammation in the skeletal muscle, and their physiological implications have not been reported to date. Muscle inflammation is widely linked to muscle damage or atrophy, both in physiological processes, such as aging^53^, and in pathophysiological processes^54^. However, whether muscle inflammation and atrophy precede one another or whether they are independent processes remains unanswered. In keeping with previous findings obtained in Opa1-depleted muscles^6^, we have shown that Mfn1-deficient skeletal muscles develop TLR9 and NFκB-dependent muscle inflammation and atrophy, resulting in reduced physical performance and enhanced IL6 response to exercise. Importantly, chronic anti-inflammatory treatment restored muscle atrophy, suggesting that the muscle inflammation triggered by Mfn1 deficiency causes the development of muscle atrophy. As a result, physical performance and the IL6-mediated systemic inflammatory response were ameliorated.

Based on our findings, we propose that the maintenance of mitochondrial dynamics is a key factor in preventing the trigger of sterile inflammatory responses characterized by mtDNA mislocation and DNA sensor activation, and that muscle inflammation induced by mitochondrial fragmentation plays a causative role in the development of muscle atrophy. Therefore, strategies to ameliorate mitochondrial dynamics could be promising as a therapeutic approach to treat inflammatory myopathies as well as other immune disorders characterized by mitochondrial dysfunction and mtDNA mislocation.

## Methods

### Animals

To generate the inducible skeletal muscle-specific deletion of Mfn1, the Mfn1^f/f^ line (obtained from MMRRC repository) was crossed with mice carrying Cre-ER under the control of the human skeletal actin tamoxifen-inducible promoter (HSA) (kindly provided by Dr. Pierre Chambon). Induction of expression of Cre was achieved by oral administration of tamoxifen-enriched chow diet (Tam400/Cre-ER Harlan), which was provided *ad libitum* for 2 weeks at the age of 12 weeks. Weight was monitored since the start of the induction. All the experiments were performed using 4–6 months-old C57BL6/J mice. Male mice were mainly used, unless otherwise specified, and mice were randomly assigned to the various groups in every experiment. Mice were kept in 12 h dark-light periods in a specific pathogen-free (SPF) animal facility and fed standard chow diet and water *ad libitum*. At indicated time, mice were anesthetized using isofluorane and euthanized by cervical dislocation. All animal experiments were done in compliance with the guidelines established by the Institutional Animal Care and Use Committee of the Barcelona Science Park and University of Barcelona (Protocol number 9279, approved by the Department of Territory and Sustainability, General Directorate of Environmental Policies and the Natural Environment, Government of Catalonia).

### Anti-inflammatory treatments in mice

TLR9 antagonist ODN2088 was administered to mice by intraperitoneal injection at a dose of 100 μg per mouse. Control mice were administered with endotoxin-free water. Mice were euthanized 48 h after injection. Sodium salicylate treatment was administered daily for 28 days by intraperitoneal injection at a 200 mg/kg dose. Control animals were administered with 1× PBS.

### Exercise until exhaustion on treadmill

Prior to the treadmill test, mice were acclimated for two consecutive days on the treadmill (BIOSEB) for 5 min of familiarization with no engagement of the treadmill belt and then, for 8 min of running at a speed of 14 cm/s and an incline of 0%. The next day, the mice rested. Then, two consecutive days of exercise until exhaustion was performed, consisting of 8 min at a starting speed of 14 cm/s, which was increased by 2 cm every 2 min until reaching the maximal speed of 46 cm/s. Exhaustion was determined when mice failed at re-engaging the belt after falling to the grid. The inclination was kept at 0% during the experiment and the intensity of the negative stimulus of the grid was set at the minimal (0.2 mA).

### Histological sample preparation and analysis

Gastrocnemius muscles were fixed overnight (O/N) at 4 °C with neutral buffered formalin (HT501128-4L, Sigma-Aldrich). Paraffin-embedded tissue sections (2–3 μm) were air-dried and further dried at 60 °C O/N.

To check tissue architecture and CSA, sections were stained with hematoxylin and eosin following standard protocol using a CoverStainer (Dako – Agilent). CSA was quantified in 120 fibers/mouse using ImageJ software.

For immunohistochemistry studies, the rat anti-F4/80, clone BM (1:100, 60 min) or the rabbit anti-IL1β (1:1000, 60 min) (Table S1) were used using the Ventana Discovery XT platform. Antigen retrieval was performed with proteinase K (S3020, Dako -Agilent) for 5 min at room temperature (RT). Blocking was done with Casein (ref: 760-219, Roche) or with Peroxidase-Blocking Solution at RT (S2023, Agilent) and 5% of goat normal serum (16210064, Life technology) mixed with 2.5% BSA diluted in wash buffer for 10 and 60 min at RT. The secondary antibody used was the OmniMap anti-Rat HRP (760–4457, Roche) or the BrightVision poly HRP-Anti-Rabbit IgG (DPVR-110HRP, ImmunoLogic). Antigen–antibody complexes were revealed with ChromoMap DAB Kit (760–159, Roche) or 3-3′-diaminobenzidine (K346811, Agilent). Sections were counterstained with hematoxylin (CS700, Dako, Agilent) and mounted with Mounting Medium, Toluene-Free (CS705, Agilent) using a Dako CoverStainer. Specificity of staining was confirmed by staining with a rat IgG (ref: 6-001-F, R&D Systems, Biotechne) or a Rabbit IgG (ab27478, Abcam) isotype controls. Brightfield images were acquired with a NanoZoomer-2.0 HT C9600 digital scanner (Hamamatsu) equipped with a ×20 objective. All images were visualized with a gamma correction set at 1.8 in the image control panel of the NDP.view 2 U12388-01 software (Hamamatsu, Photonics, France).

### Blood collection and quantifications in plasma

Blood samples were collected in EDTA-coated microvettes® (Sarstedt) and centrifuged at 4 °C, 2500 rpm for 30 min. Plasmas were collected and kept at −20 °C, avoiding repetitive freezing and thawing cycles. Plasma levels of IL6 (Merck), TNFα (Abnova), FGF21 (Abcam), and creatine kinase activity (Merck) were quantified following the manufacturer’s instruction.

### Cell culture, gene manipulation, and treatments

C2C12 myoblasts (ATCC, catalog number CRL-1772) and HeLa cell lines (routinely used in our laboratory) were grown in DMEM 4.5 g/L Glucose, 1× Pen/Strep (complete DMEM) supplemented with 10% fetal bovine serum (FBS). C2C12 myoblasts were differentiated into myotubes by replacing the growing media with differentiation media containing DMEM 4.5 g/L Glucose, 1× Pen/Strep (complete DMEM) supplemented with 2% horse serum (HS) for 4 days. C2C12 cells were used until passage 15, given that longer passages affect their differentiation capacity.

To generate stable KD C2C12 cell lines, pLKO.1 plasmids (Table S2) encoding for shRNAs were delivered to wild-type (WT) C2C12 myoblasts using lentiviral infection in order to allow their stable expression. Cell lines were selected and maintained using 1 μM puromycin. C2C12 stably expressing Scramble shRNA (Scr) was used in parallel in all the experiments as negative controls.

Cells were treated with TLR9 antagonist ODN2088 or cGAS inhibitor Ru.521 at 1 μM for 24 h, with ddC at 40 μM for 72 h, or Bafilomycin A1 at 200 nM for 16 h.

Acute knockdowns in myoblasts were achieved by MISSION® predesigned siRNA transfection (Table S3) using a final siRNA concentration of 10 nm with lipofectamine 2000 and allowing protein downregulation for 24 h. The negative control corresponds to the transfection of a universal negative control siRNA (SIC001).

Transduction of adenoviruses was performed differently depending on the targeted cell type. Myotubes at day 4 of differentiation were transduced for 16 h with control adenoviruses, adenoviruses containing miRNAs against Mfn1 or Mfn2 (MOI 100)^9^, and adenoviruses containing shRNAs against Fis1 (MOI 200, Vector Biolabs) or Drp1 (MOI 200, Vector Biolabs), and processed 2 days after transduction. Myoblasts were transduced with adenoviruses containing overexpressing vectors for LacZ, Drp1, or a dominant-negative form of Drp1, Drp1K48A, at an MOI of 200^55^.

To generate the MFN1-HA HeLa cell line, WT cells were transfected with 9 µg/ml PEI in NaCl 150 mM. A pSpCas9(BB)−2A-GFP (PX458) plasmid (#48138, Addgene) containing the DNA sequence of a specific guide RNA for *MFN1* (5′ CCAAAGCAATCTCTATTGTT 3′) was introduced to the cells, along with single-stranded oligodeoxynucleotide homologous to the sequence of *MFN1* that contained the sequence of the HA-tag (5′AATTTTACTAAGCAGTTTCTACCTTCAAGCAATGAAGAATCCTACCCATACGATGTTCCAGATTACGCTTAACAATAGAGATTGCTTTGGTGACCATGATAGGAGGAAAC3′). 48 h after transfection, cells expressing GFP were isolated by single-cell sorting using a BD FACSAria™ Fusion Flow Cytometer and expanded until monoclonal cell lines were obtained. The presence or absence of HA was analyzed by western blot.

### Pro-inflammatory cytokine quantification in cultured media

IL1β and IFNβ concentration in culture media of stable KD myoblasts was measured using the IL1β (mouse) ELISA kit (Sigma, RAB0274-1KT) and the VeriKine-HS (mouse) IFNβ ELISA Kit (PBL Assay Science, 42410-1), respectively, following the manufacturer’s instruction. Prior to the assay, stable KD myoblasts were plated in 10 cm^2^ dishes and allowed growth until reaching 90% confluency. 10 mL of culture mediums were collected and concentrated down to 400 uL using the Vivaspin 6 centrifugal concentrators (Sigma, Z614483), following the manufacturer’s instructions.

### Immunofluorescence (IF) and super-resolution microscopy image processing

Stable KD cells were fixed in 4% paraformaldehyde (PFA) in PBS for 20 min and washed for 10 min with PBS. Cells usually permeabilized in 0.1% triton X-100 and 3% FBS in PBS for 20 min and then coverslips were incubated for 10 min in 0.05% Saponin and 2% FBS in PBS (buffer A). Coverslips were incubated in primary antibody diluted in buffer A (1:400) for 60 min, washed with buffer A, incubated in secondary antibody diluted buffer A (1:800) for 60 min and washed with buffer A. Cells were then washed with PBS before mounting the coverslips on microscope slides with Flouromount (Sigma). Confocal images were obtained using the super-resolution Airyscan detector of ZEISS Elyra 7 microscope at x64 magnification and 1.4NA. Used primary antibodies are specified in Table S1. Mitochondrial staining was achieved incubating cells with Mitotracker Deep Red (ThermoFisher) at 50 nM for 30 min prior fixation. Secondary antibodies used were Alexa Fluor 488, Alexa Fluor 568, and Alexa Fluor 647. Image processing and quantifications were performed using ImageJ software^56^. All co-distribution quantifications were performed with JACoP plugin^57^. Quantification of the branch length of mitochondria was performed with MiNA plugin. All quantifications were done on stacks of images. 3D reconstructions were generated using IMARIS software from images obtained using the super-resolution Airyscan detector of ZEISS Elyra 7 microscope at ×100 magnification and 1.4NA.

### Subcellular fractionation in cells

Stable KD cell pellets were incubated in digitonin containing buffer (150 mM NaCl, 50 mM Hepes, and 50 μg/mL digitonin (Sigma)) end-over-end for 10 min at 4 °C. Then, homogenates were centrifuged at 980 × *g* for 4 min at 4 °C. Supernatants were again centrifuged at 17,000 × *g* for 10 min at 4 °C and to allow removal of any remaining cellular debris. Supernatants were kept as cytosolic fractions and used for subsequent genomic and mitochondrial DNA analysis. Purity of the fractions was validated through western blotting of a lysosomal marker (LAMP1), a mitochondrial marker (TIMM23), and a cytosolic marker (Tubulin).

### Immunoprecipitation and mass spectrometry analysis (IP-MS)

HeLa WT and MFN1-HA cells were used. From each cell line, 1.4 × 10^8^ cells were recovered from 15 cm^2^ plates. Pelleted cells were resuspended in 2 pellet volumes of lysis buffer (50 mM Tris pH 7.4, 150 mM NaCl, 5 mM EDTA, 1% Digitonin, Roche Complete EDTA Free protease inhibitors, Merck Cocktail set IV phosphatase inhibitors). Cells were disrupted manually in a Dounce homogenizer after 20 strokes. Lysates were transferred to 2 ml tubes and incubated on ice for at least 15 min. Samples were centrifuged at maximum speed for 15 min at 4 °C. Supernatants were incorporated into protein LoBind tubes (Eppendorf). A fraction of these samples was kept as an input control. Samples were incubated with 40 µl of HA-dynabeads at 4 °C in rotation for 12 h. Next, a magnetic rack was used to separate the beads from the supernatant. A fraction of the supernatant was kept as an unbound control and the rest of the supernatant was discarded. Beads were washed five times in 1 ml ice-cold wash buffer (50 mM Tris pH 7.4, 150 mM NaCl, 5 mM EDTA, 0.1% Digitonin, Roche Complete EDTA Free protease inhibitors, Merck Cocktail set IV phosphatase inhibitors). Beads were eluted in 2× Laemmli buffer in a volume equal to the volume of beads and boiled for 5 min at 95 °C. Next, samples were run on a 0.75 mm SDS-PAGE gel until they passed the stacking gel. The gel was stained with InstantBlue® Coomassie Protein Stain (Abcam) and bands were cut.

Stained electrophoretic protein bands were reduced with DTT 10 mM for 45 min at 56 °C and alkylated for 30 min in the dark with IAA 50 mM. Then, in-gel digestion was performed with trypsin (0.1 µg/µL) in 50 mM NH_4_HCO_3_ at 37 °C overnight. The digestion was stopped by adding formic acid. Peptides were extracted with 100% acetonitrile and completely evaporated. Samples were reconstituted in 20 µL of 3% Acetonitrile (ACN) and 1% formic acid (FA) aqueous solution for MS analysis.

The nano-LC-MS/MS set up was as follows. Digested peptides were diluted in 1% FA, 3% ACN. Samples were loaded to a 300 µm × 5 mm, C18 PepMap100 (Thermo Scientific) at a flow rate of 15 µl/min using a Thermo Scientific Dionex Ultimate 3000 chromatographic system (Thermo Scientific). Peptides were separated using a C18 analytical column (NanoEase MZ HSS T3 column, 75 µm × 250 mm, 1.8 µm, 100 Å, Waters) with a 90 min run, comprising three consecutive steps with linear gradients from 3 to 35% B in 60 min, from 35 to 50% B in 5 min, and from 50 to 85% B in 2 min, followed by isocratic elution at 85% B in 5 min and stabilization to initial conditions (A = 0.1% FA in water, B = 0.1% FA in CH3CN). The column outlet was directly connected to an Advion TriVersa NanoMate (Advion) fitted on an Orbitrap Fusion Lumos™ Tribrid (Thermo Scientific). The mass spectrometer was operated in a data-dependent acquisition (DDA) mode. Survey MS scans were acquired in the orbitrap with the resolution (defined at 200 m/z) set to 120,000. The lock mass was user-defined at 445.12 m/z in each Orbitrap scan. The top speed (most intense) ions per scan were fragmented by CID and detected in the Ion Trap. The ion count target value was 400,000 for the survey scan and 50,000 for the MS/MS scan. Target ions already selected for MS/MS were dynamically excluded for 15 s. Spray voltage in the NanoMate source was set to 1.60 kV. RF Lens were tuned to 30%. Minimal signal required to trigger MS to MS/MS switch was set to 5000 and activation Q was 0.250. The spectrometer was working in positive polarity mode and singly charge state precursors were rejected for fragmentation.

A database search was performed with Proteome Discoverer software v2.3 (Thermo) using Sequest HT search engine, SwissProt Human release 2019_01 and Contaminants database. Search was run against targeted and decoy database to determine the false discovery rate (FDR). Search parameters included Trypsin (and Chymotrypsin) enzyme, allowing for two missed cleavage sites, carbamidomethyl in cysteine as static modification; methionine oxidation and acetylation in N-terminal as dynamic modifications. Peptide mass tolerance was 10 ppm and the MS/MS tolerance was 0.6 Da. Peptides with an FDR < 1% were considered as positive identifications with a high confidence level.

For the quantitative analysis, contaminant identifications were removed and unique peptide spectrum matches of protein groups identified with Sequest HT and Andromeda were analyzed with SAINTexpress-spc v3.11^58^. SAINTexpress compares the prey control spectral counts with the prey test spectral counts for all available replicates. For each available bait and for each available replicate, the maximum count result between PD and MQ was taken as prey count. High confidence interactors were defined as those with Bayesian false discovery rate BFDR ≤ 0.05.

### Immunoprecipitation

HeLa WT and stable Scr or Mfn1KD myobalsts were grown in six 15 cm^2^ plates and transfected with 10 μg of FLAG-RAB5C (HG15817-NF, SinoBiological) per plate. 48 h after transfection, cells were collected and solubilized in lysis buffer (Tris HCl 50 mM, NaCl 150 mM, EDTA 5 mM, 1% digitonin) for 20 min in ice to allow homogenization. Samples were centrifuged at 21,000 × *g* for 10 min at 4 °C and supernatants were kept. A fraction of the lysate of each sample was kept as an input control. 20–30 μL of magnetic anti-FLAG magnetic beads (Sigma) were incubated with 2.5–5 mg of protein from lysates O/N at 4 °C. Unbound supernatant was separated from the beads using a magnetic rack (Applied Biosystems Dynal MPC-S Dynabeads). A fraction of each supernatant was kept as an unbound or flowthrough control and the rest was discarded. Beads were washed three times in wash buffer (Tris HCl 50 mM, NaCl 150 mM, EDTA 5 mM, 0.1% digitonin). Elution was achieved by incubating the beads with 20 μL of 1:50 FLAG peptide (Sigma) for 15 min in ice. All input, unbound (flowthrough, FT) and immunoprecipitated fractions were mixed with 4× LSB + DTT and loaded in a western blot.

### Protein extraction and western blotting (WB)

Cell culture homogenates for western blot analyzes from were obtained by collecting the cells in ice-cold PBS 1×. Cells were homogenized with a 25G-syringe in lysis buffer (50 mM Tris (pH 7.5), 150 mM NaCl, 1 mM EDTA, 2 mM sodium orthovanadate, 100 mM NaF, 20 mM sodium pyrophosphate, 1% NP-40, and protease inhibitors Mini protease tablet (Roche)) and centrifuging them at 700 × *g* for 10 min and 4 °C to remove nuclei, cell debris and floating cells. Mouse skeletal muscle, liver, kidney and heart tissues were homogenized in lysis buffer with a Minibeadbeater (Biospec) twice for 30 sec, incubated for 1 h at 4 °C in an orbital shaker, and then centrifuged for 15 min at 10,000 × *g*. Supernatants were aliquoted and kept at −20 °C.

Proteins from total homogenates were solved in 10%, 12.5%, or 15% acrylamide gels for SDS-PAGE and transferred to Immobilon membranes (Millipore). Primary antibodies used are specified in Table S1. Secondary antibodies used included donkey anti-mouse HRP (715-035-150, Jackson Laboratories) and donkey anti-rabbit HRP (711-035-152, Jackson Laboratories), donkey anti-rat HRP (760–4457, Roche), goat anti-mouse DyLight 800 (SA535521, Invitrogen), goat anti-mouse IRDye 680LT (35518, Licor Biosciences), goat anti-rabbit DyLight 800 (SA535571, ThermoFisher), goat anti-rabbit Alexa Fluor 680 (A-21076, ThermoFisher).

### Determination of mitochondrial parameters using flow cytometry

Stable KD myoblasts were incubated with the indicated probe according to the cognate assay, washed twice with PBS 1× and trypsinized. Fluorescence was quantified by flow cytometry in a Gallios cytometer (Beckman Coulter). Mitochondrial membrane potential was measured with TMRE (ThermoFisher) at 50 nM for 30 min, mitochondrial mass with Mitotracker Green or Deep Red (ThermoFisher) at 50 nM for 1 h and mitochondrial superoxide with MitoSOX (ThermoFisher) at 50 nM for 30 min.

### Determination of mitochondrial oxygen consumption

Respiration of Scr and KD myoblasts was performed using the XF-24 Extracellular Flux Analyzer (Seahorse Bioscience, Agilent Technologies). Cells were seeded in XF-24 plates. At a confluency of 80%, respiration was measured in the presence of 5.5 mM glucose, 1 mM sodium pyruvate, and 2 mM glutamine (basal respiration). Then 5 µM oligomcyin (leak), 0.5 µM CCCP (maximal respiration) and 1 µM rotenone, and 1 µM Antimycin A (non-mitochondrial respiration) were subsequently added.

### Mitochondrial DNA content

Genomic DNA was purified from cultured cells and cytosolic fractions by using Genelute Mammalian Genomic DNA Kit (Sigma) according to the manufacturer’s instructions. DNA was amplified with specific oligodeoxynucleotides for mitochondrial DNA or nuclear DNA. Specific primers to amplify mouse mitochondrial genes specified in Table S4. Mitochondrial DNA content was calculated using *Gapdh* amplification as a reference for nuclear DNA content.

### Transmission electron microscopy and immunolabelling (IG)

For mitochondrial morphology assessment in vivo, quadriceps muscles were dissected and cut into pieces of ~1 mm^3^. The pieces were fixed with 2% paraformaldehyde and 2.5% glutaraldehyde in phosphate buffer (PB) and kept in the fixative for 24 h at 4 °C. Samples were then washed with the same buffer and post-fixed with 1% osmium tetroxide in the same buffer containing 0.8% potassium ferricyanide at 4 °C. Dehydration was performed in acetone, infiltration with Epon resin for 2 days, and then samples were embedded in the same resin orientated for longitudinal sectioning and polymerized at 60 °C for 48 h. Semi-thin sections were made in order to corroborate that the orientation was satisfactory under the light microscope. Ultrathin sections were obtained using a Leica Ultracut UC6 ultramicrotome (Leica Microsystems, Vienna, Austria) and mounted on Formvar-coated copper grids. They were stained with 2% uranyl acetate in water and lead citrate. Sections were then observed undera JEM-1010 electron microscope (Jeol, Japan) equipped with a CCD camera SIS Megaview III and the AnalySIS software.

For immunolabelling, samples were prepared at the Electron Cryomicroscopy Unit from the CCiTUB, University of Barcelona. Scr and Mfn1KD myoblasts were washed twice with 0.1 M PB at room temperature. For fixation, cells were fixed in 4% paraformaldehyde 0.1% glutaraldehyde in 0.1 M PB at room temperature for 60 min. Then, they were kept in 2% paraformaldehyde in 0.1 M PB till at 4 °C overnight. Samples were cryoimmobilized in 15% dextran in 10 mM PBS as a cryoprotectant using a Leica HPM100 High-Pressure Freezer (Leica Microsystems, Vienna, Austria). Planchettes containing the frozen samples were transferred to cryotubes containing 0.5% uranyl acetate (EMS, Hatfield, USA) in acetone under liquid nitrogen and were freeze substituted at −90 °C for 80 h in an EM AFS2 (Leica Microsystems, Vienna, Austria). Samples were warmed up to −40 °C at 5 °C/h slope and kept at −40 °C. They were rinsed with acetone and infiltrated in Lowicryl HM23 resin (EMS, Hatfield, USA) at −40 °C. Samples were polymerized under UV light: at –40 °C for 24 h, during the warming up at 5 °C/h slope until 22 °C and at 22 °C for 48 h. Sections of 60 nm in thickness were obtained using a UC6 ultramicrotome (Leica Microsystems, Vienna, Austria). They were washed sequentially in 10 mM PBS, 10 mM glycine, and 10 mM PBS. Then they were incubated on drops of 5% bovine serum albumin (BSA) in 10 mM PBS for 20 min and they were changed to 1% BSA in 10 mM PBS drops, followed by the incubation with SdhA antibody (1:20) and Rab5 antibody (1:4) (Table S1) in 10 mM PBS for 1 h. Then, they were washed in 0.25% Tween 20 in 10 mM PBS and they were changed to 1% BSA in 10 mM PBS, followed by the incubation in anti-mouse 12 nm 1:30 (Jackson) and anti-rabbit 18 nm 1:30 (Jackson) in 1% BSA 10 mM PBS for 30 min. Samples were washed in 10 mM PBS, incubated in 1% glutaraldehyde in PBS for 5 min, and rinsed in milliQ water. As a negative control for non-specific binding of the colloidal gold-conjugated antibody, the primary polyclonal antibody was omitted. Sections were stained with 2% uranyl acetate and lead citrate and were observed in a Tecnai™ Spirit TWIN microscope (FEI, Eindoven, The Netherlands) equipped with a tungsten cathode from the Electron Cryomicroscopy Unit from the CCiTUB. Images were acquired at 120 kV with a CCD Megaview 1k × 1k.

### RNA extraction and real-time-qPCR

RNA from cells was extracted by using PureLink RNA Mini Kit (Invitrogen) following the manufacturer’s instructions. RNA from quadriceps muscles was isolated by using the Trizol reagent followed by purification with PureLink RNA Mini Kit (Invitrogen). An intermediate step of DNase treatment was performed in RNA isolation from tissues to improve purity. RNA was reverse-transcribed by using the Q-Script cDNA SuperMix (QuantaBio). Quantitative real-time PCR was performed using the QuantStudio 6 Real-Time PCR system (ThermoFisher) and the SYBR® Green PCR Master Mix (Applied Biosystems). All measurements were normalized to *b-actin* or *36b4*. The primers used are specified in Table S5.

### Statistical analysis

The data presented here was collected using Excel 2016 and was analyzed GraphPad Prism version 8.0.1 and 9.4.1 for Windows, GraphPad Software, San Diego, California USA, www.graphpad.com. An appropriate normality test was performed to assess whether the data fit a Gaussian distribution. Statistical significance was determined using the Student *t* test or analysis of variance with an appropriate post hoc *t* test. Outliers were detected using Grubbs’ test. Data are presented as mean ± SEM. Significance was established at *p* < 0.05.

### Graphics

Data plots were generated with GraphPad Prism version 8.0.1 and 9.4.1 for Windows, GraphPad Software, San Diego, California USA, www.graphpad.com. The graphics shown in Figs. 1a, c, 2b, d, 5a, Supplementary Fig. 1f, 2a–h, 6h, and 8g were generated with ©BioRender – biorender.com (Toronto, Canada).

### Reporting summary

Further information on research design is available in the Nature Portfolio Reporting Summary linked to this article.

## Supplementary information

Supplementary information

Reporting Summary

## Data Availability

All data generated or analyzed during this study are included in this published article (and its supplementary information and Source Data files). Databases used include SwissProt Human release 2019_01 and Contaminants databases. The mass spectrometry proteomics data have been deposited to the ProteomeXchange Consortium via the PRIDE^59–61^ partner repository with the dataset identifier PXD037935. Source data are provided with this paper.

## Source data

Source data file
