# Supplementary material for: Disruption of mitochondrial dynamics triggers muscle inflammation through interorganellar contacts and mitochondrial DNA mislocation
Source: Nat Commun. 2023 Jan 6;14:108. doi: 10.1038/s41467-022-35732-1 (PMC9822926; doi:10.1038/s41467-022-35732-1)
Supplement: Supplementary file 1 — Supplementary information [file 41467_2022_35732_MOESM1_ESM.pdf]

DISRUPTION OF MITOCHONDRIAL DYNAMICS TRIGGERS MUSCLE INFLAMMATION THROUGH INTERORGANELLAR CONTACTS AND MITOCHONDRIAL DNA MISLOCATION

Supplementary figures and legends

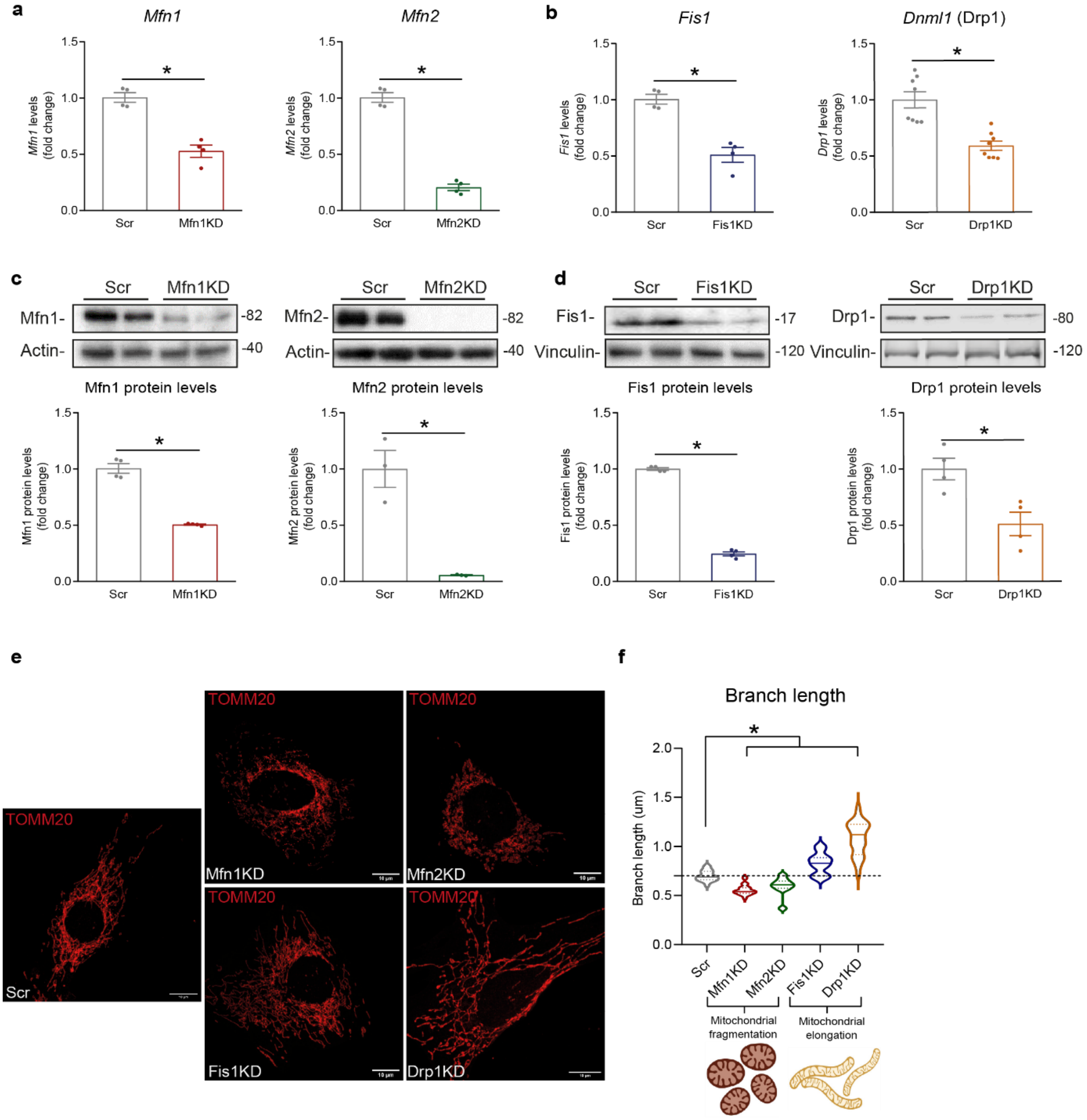

Supplementary Fig. 1 (**a**) mRNA levels of *Mfn1* and *Mfn2* in Mfn1-deficient or Mfn2-deficient myoblasts, respectively (n=4). (**b**) mRNA levels of *Fis1* and *Drp1* in Fis1-deficient or Drp1-deficient myoblasts, respectively (n=4-8). (**c**) Mfn1, Mfn2 and housekeeping (Actin) representative immunoblots in Mfn1-deficient or Mfn2-deficient myoblasts, respectively (n=3-4), and band quantifications. (**d**) Fis1, Drp1 and housekeeping (Vinculin) in Fis1-deficient or Drp1-deficient myoblasts, respectively (n=4), and band quantifications. (**e**) Representative immunostainings targeting TOMM20 (red) in all cell lines (n=20 images per condition) (Scale bar 10 µm). (**f**) Quantification of the branch length of the mitochondrial networks per cell line (n=20). (**a** - **d**) Two-sided Students' T-test, (**f**) One-Way ANOVA test and post-hoc t tests.

Data are expressed as mean of n independent experiments ± SEM. *p vs Scr <0.05. (**f**) Created with BioRender.com. (**a - d** and **f**) Source data is provided in the Source Data File.

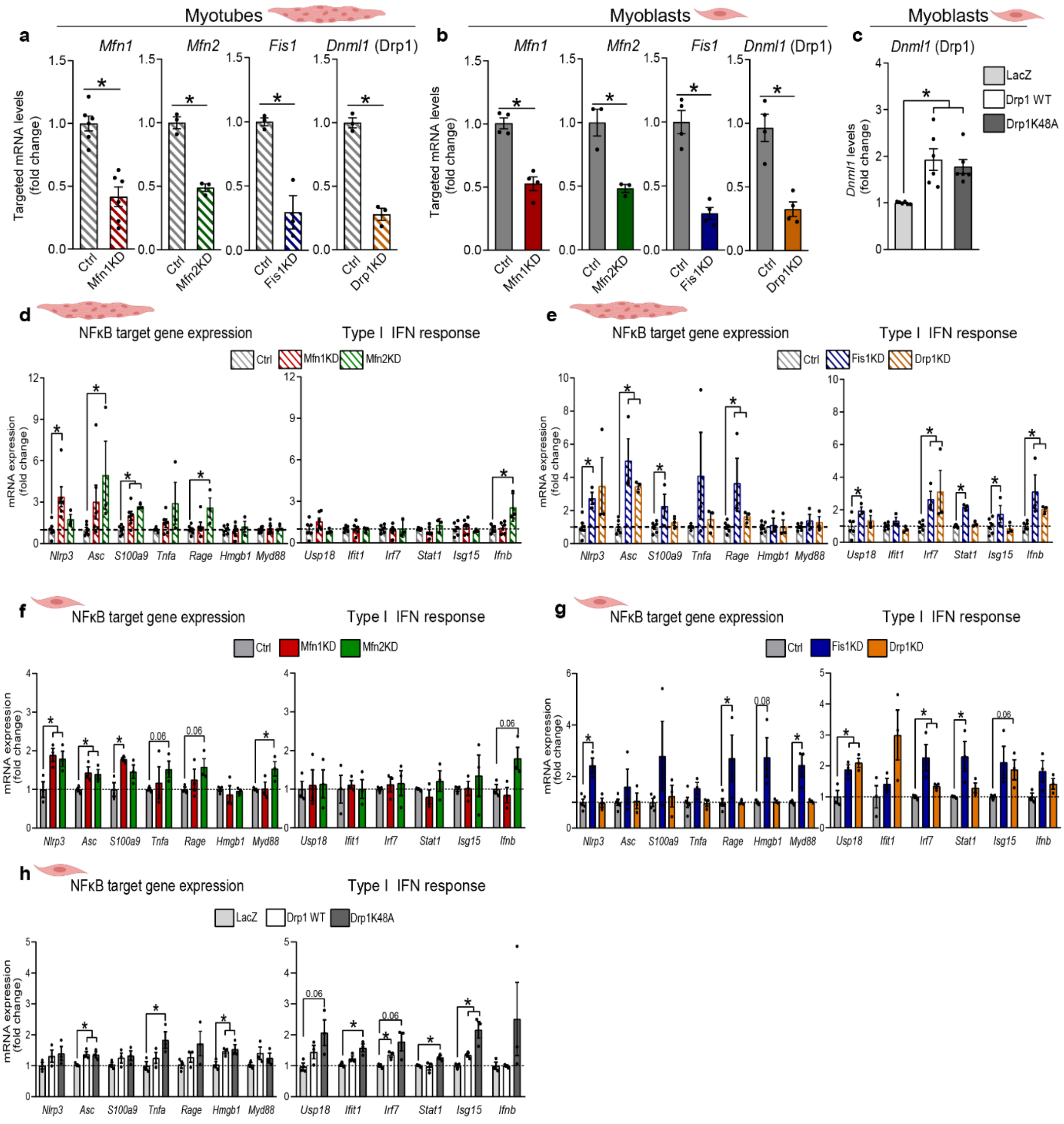

Supplementary Fig. 2 (**a**) Validation of transient KD of mitochondrial dynamics proteins in (**a**) differentiated myotubes and (**b**) myoblasts (n=3-6). (**c**) Validation of Drp1 overexpression in myoblasts (n=6). Inflammatory profile in (**d**) Ctrl, Mfn1KD and Mfn2KD myotubes or (**e**) Ctrl, Drp1KD and Fis1KD myotubes (n=3-6). Inflammatory profile in myoblasts transfected with (**f**) siCtrl, siMfn1 and siMfn2, or (**g**) siCtrl, siFis1 and siDrp1 (n=3). (**h**) Inflammatory profile in myoblasts transduced with overexpression vectors of LacZ, wildtype Drp1 and negative dominant form of Drp1 (K48ADrp1) (n=3). (**a**, **b, d - h**) Two-sided Students' T-test, per gene in d - h, (**c**) One-way ANOVA test. Data are expressed as mean of n independent experiments ± SEM. *p vs Ctrl/LacZ <0.05. (**a - h**) Created with BioRender.com. (**a - h**) Source data is provided in the Source Data File.

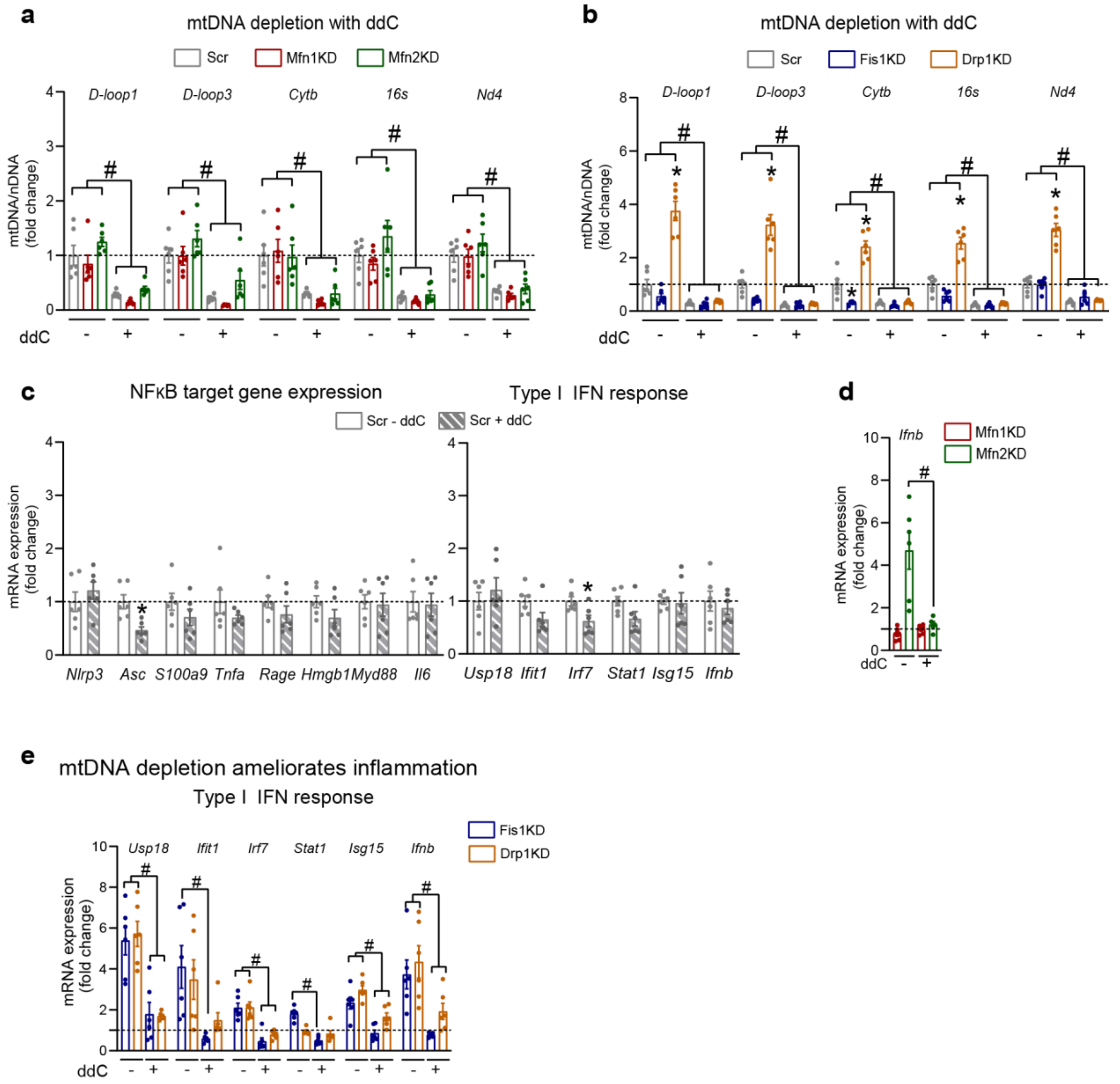

Supplementary Fig. 3. mtDNA abundance upon ddC treatment (40uM, 72h) in (**a**) Scr, Mfn1 or Mfn2-deficient myoblasts or (**b**) Scr, Fis1 or Drp1-deficient myoblasts (n=6). (**c**) Inflammatory profile of Scr myoblasts treated with ddC (n=6). (**d**) *Ifnb* expression in Mfn1 or Mfn2KD myoblast upon ddC treatment (n=6). (**e**) Type I IFN response in Fis1- and Drp1KD myoblasts upon ddC treatment. (**a** - **e**) Two-sided Students' T-test per gene. Data are expressed as mean of n independent experiments ± SEM. *p vs Scr - ddC <0.05 in and #p vs. cognate KD - ddC <0.05. (**a - e**) Source data is provided in the Source Data File.

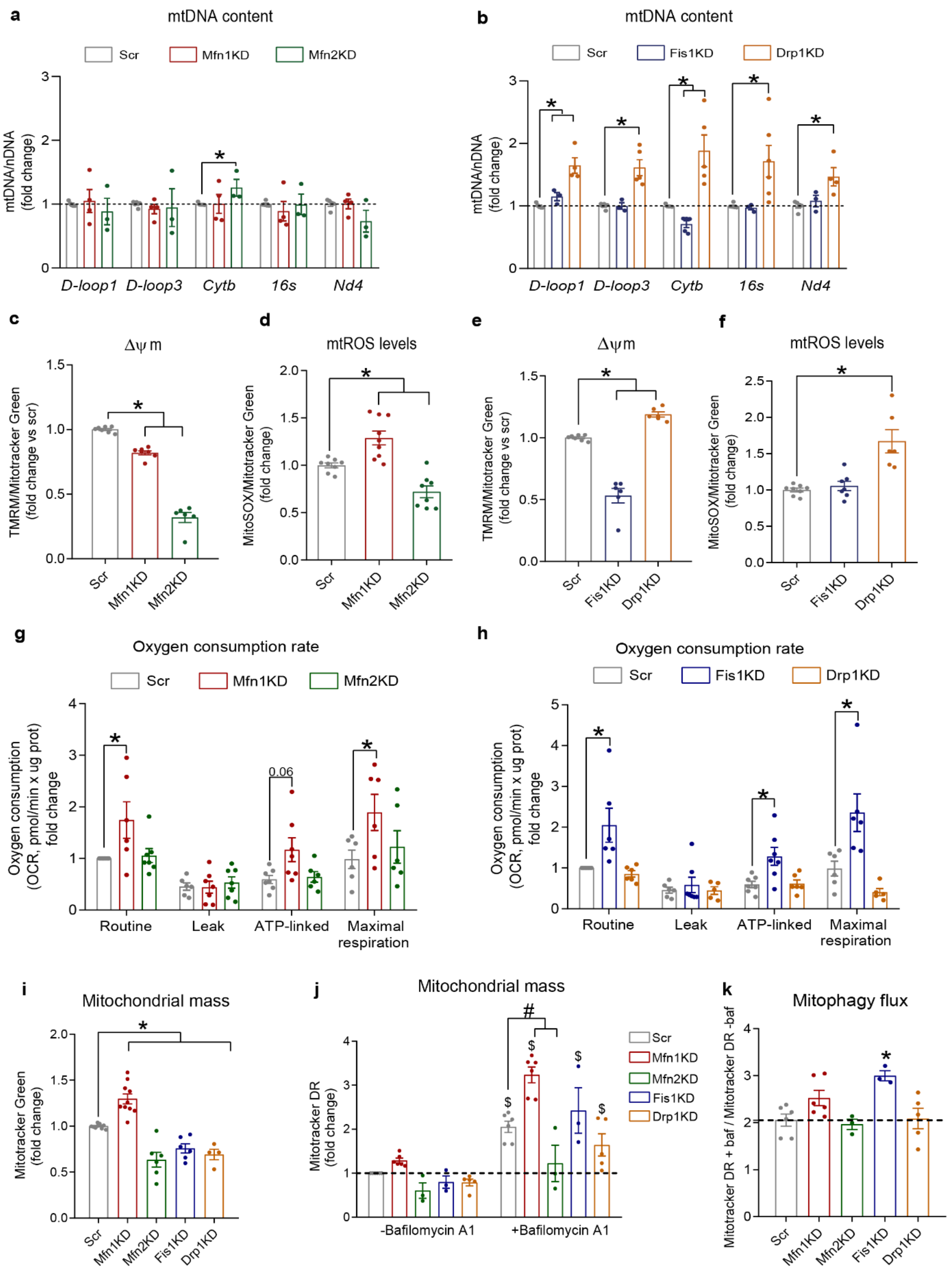

Supplementary Fig. 4. mtDNA abundance in (**a**) Scr, Mfn1 or Mfn2-deficient myoblasts (n=3-4) or (**b**) Scr, Fis1 or Drp1-deficient myoblasts (n=3-4). (**c**) TMRM relative to Mitotracker Green in Scr, Mfn1 or Mfn2-deficient myoblasts (n=6). (**d**) MitoSOX relative to Mitotracker Green in Scr, Mfn1 or Mfn2-deficient myoblasts (n=8). (**e**) TMRM relative to Mitotracker Green in Scr, Fis1 or Drp1-deficient myobalsts (n=5-6). (**f**) MitoSOX relative to Mitotracker Green in Scr,

Fis1 or Drp1-deficient myoblasts (n=6-7). Oxygen consumption rates in (**g**) Scr, Mfn1 or Mfn2-deficient myoblasts (n=6-7) or (**h**) Scr, Fis1 or Drp1-deficient myoblasts (n=5-7). (**i**) Mitotracker Green values in all cell lines. (**j**) Mitotracker Deep Red values in all cell lines with or without Bafilomycin A1 (200nM, 16h) and ratio of the signal with vs without Bafilomycin A1 as a measure of the mitophagic flux. (**a**, **b**) Two-sided Students' T-test per gene. (**c – f, i, k**) One-way ANOVA test and post-hoc t tests, (**g, h**, **j**) Two-way ANOVA test and post-hoc t tests. Data are expressed as mean of n independent experiments ± SEM. *p vs Scr <0.05 in (**a – i, k**); *p vs Scr - Bafilomycin A1 <0.05, # $p vs Scr + Bafilomycin A1 <0.05, vs Scr/cognate KD – Bafilomycin A1 <0.05 in (**j**). Source data is provided in the Source Data File.

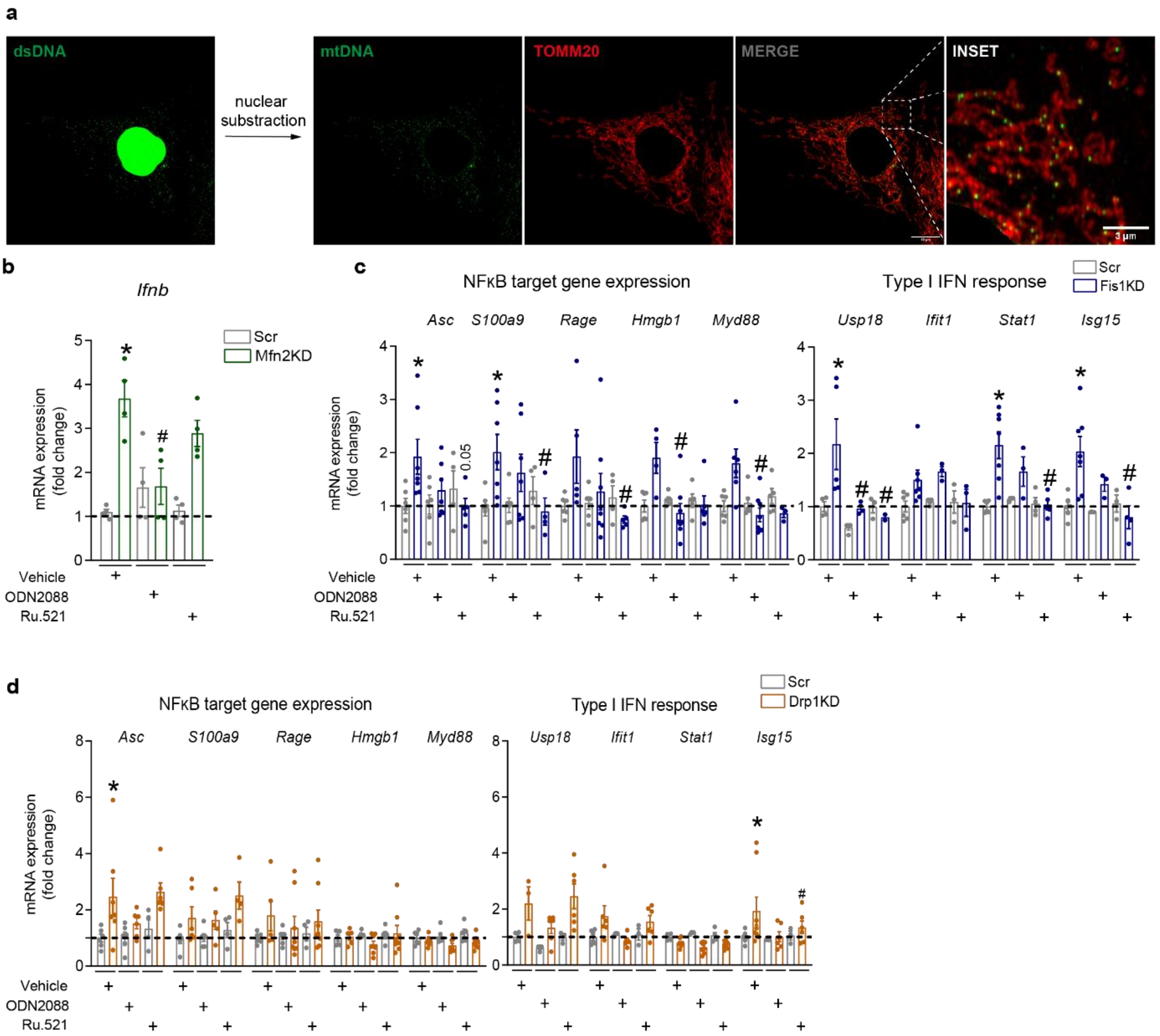

Supplementary Fig. 5 (**a**) Representative immunostaining of dsDNA (green) with nuclear subtraction (mtDNA) and TOMM20 (red) in a Scr myoblast. (**b**) *Ifnb* mRNA levels upon ODN2088 or Ru.521 (1uM, 24h) treatment in Scr and Mfn2-myoblasts (n=4). NFκB target gene and type I IFN response gene levels upon ODN2088 or Ru.521 treatment in (**c**) Scr and Fis1-deficient myoblasts, and (**d**) Scr and Drp1-deficient myoblasts (n=3-7). (**b**) One-Way ANOVA test, (**c, d**) Two-way ANOVA test and post-hoc t tests. Data are expressed as mean of n independent experiments ± SEM. *p vs Scr + vehicle <0.05 and #p vs. cognate KD + vehicle <0.05 in (**b** – **d**). (**b - d**) Source data is provided in the Source Data File.

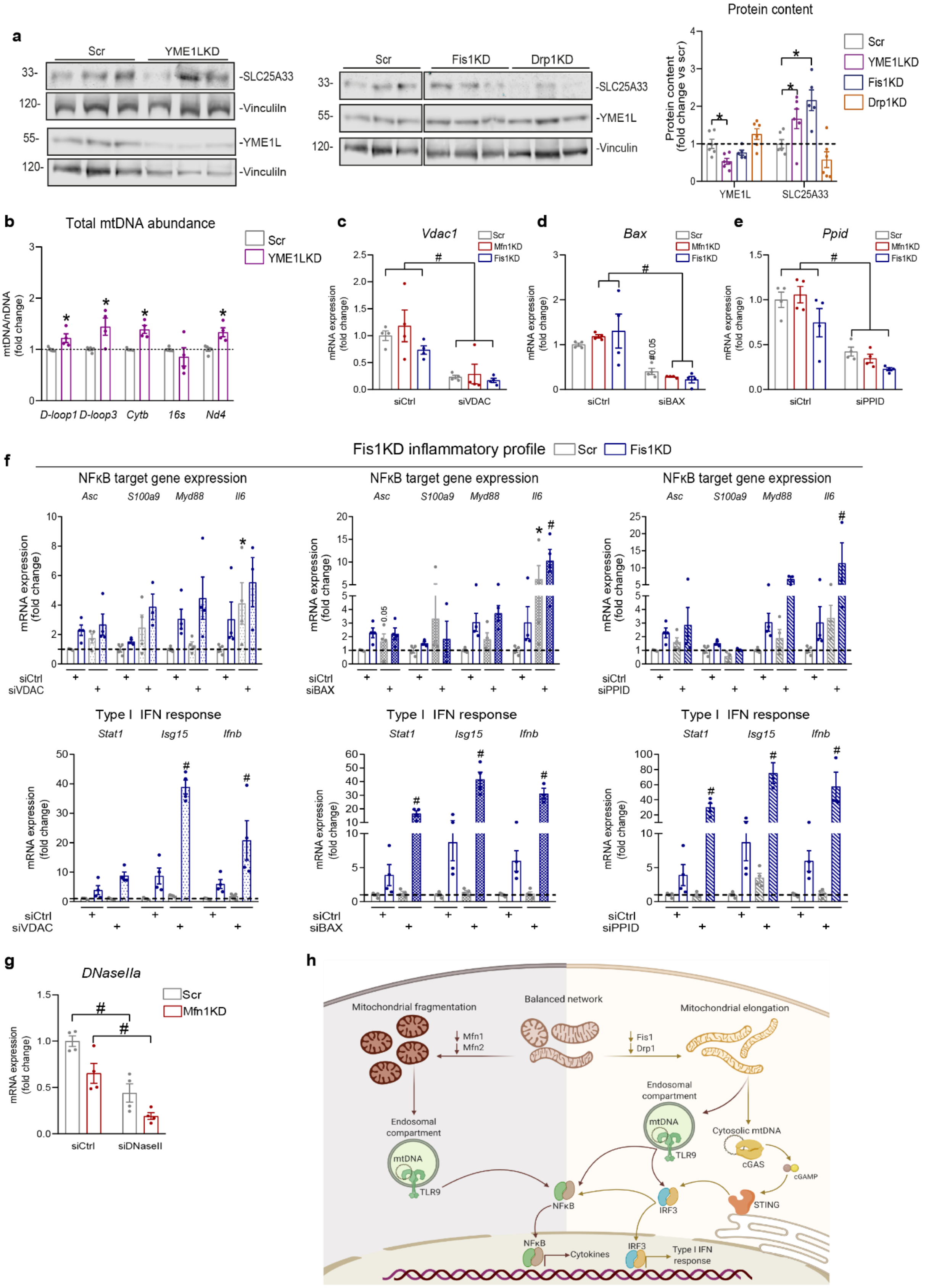

Supplementary Fig. 6 (**a**) SLC25A33, YME1L and Vinculin representative immunoblots and band quantification of SLC25A33 and YME1L in myoblasts deficient of YME1L, FIS1 or DRP1

compared to Scr. (**b**) mtDNA abundance in YME1L-deficient myoblast (n=4) mRNA of (**c**) *Vdac1*, (**d**) *Bax*, and (**e**) *Ppid* upon siCtrl, siVDAC, siBAX and siPPID transfection, respectively, in Scr, Mfn1 or Fis1-depleted myoblasts (n=4). (**f**) Inflammatory profile upon *Vdac1*, *Bax* or *Ppid* acute downregulation in Scr and Fis1KD myoblasts (n=4). (**g**) *DNaseIIa* mRNA levels upon siDNaseII tranfection in Scr and Mfn1-deficient myoblasts (n=4). (**h**) Graphical abstract showing the molecular mechanisms described coupling opposite mitochondrial morphologies and the trigger of intracellular inflammatory responses. (**a**) One-way ANOVA test, (**b**) Two-sided Students' T-test per gene, (**c** - **g**) Two-way ANOVA test and post-hoc t tests. Data are expressed as mean of n independent experiments ± SEM. *p vs Scr <0.05 in (**a**, **b**), *p vs Scr + siCtrl <0.05 and #p vs Scr/cognate KD + siCtrl <0.05 in (**c** – **g**). (**h**) Created with BioRender.com. (**a - g**) Source data is provided in the Source Data File.

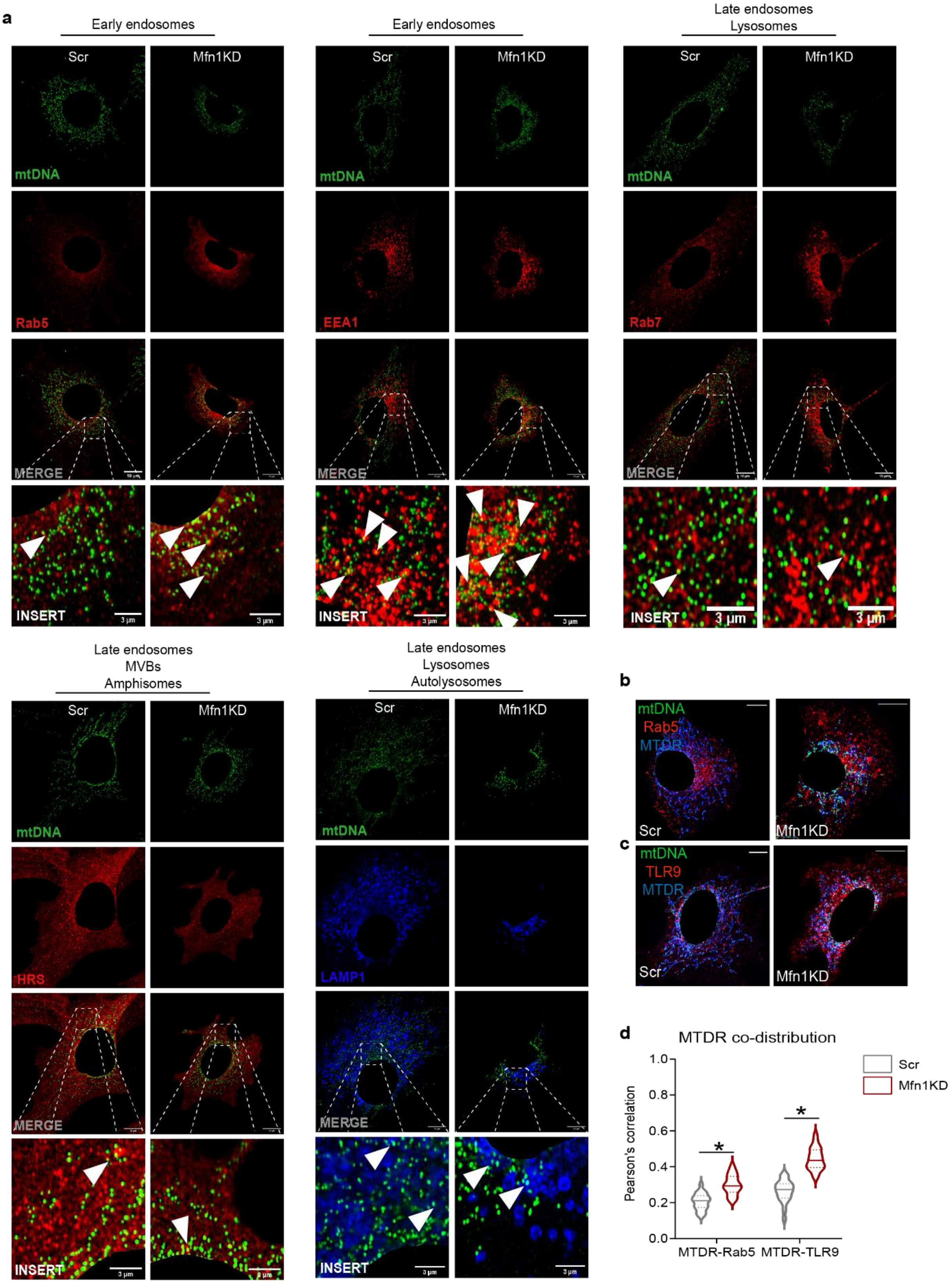

Supplementary Fig. 7. (**a**) Representative immunostainings of dsDNA (green) with subtraction of the nuclear signal (mtDNA), with endosomal markers Rab5, EEA1, Rab7, HRS (red) or LAMP1 (blue) in Scr and Mfn1-deficient myoblasts (Scale bar 10 µm in MERGE and 3 µm in INSET) (n=20 images per condition). (**b**) Quantification of the Pearson's correlation

between Mitotracker Deep Red (MTDR) and Rab5 or TLR9 in Scr and Mfn1-myoblasts (n=20) from (**c** and **d**) images used for 3D reconstructions (Scale bar 10 µm). (**d**) Two-sided Students' T-test. Data are expressed as mean ± SEM. *p vs Scr <0.05. (**d**) Source data is provided in the Source Data File.

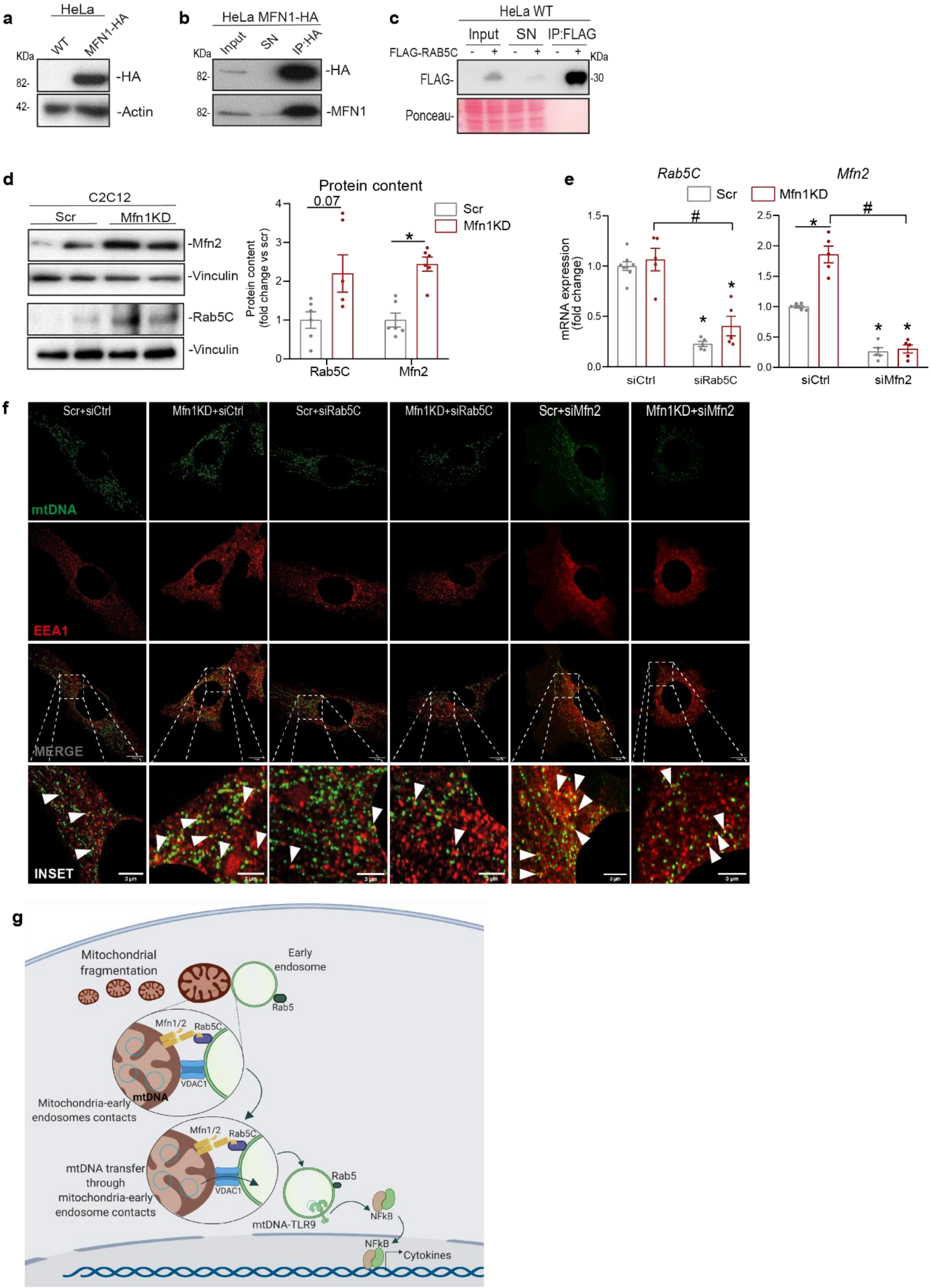

Supplementary Fig. 8 (**a**) HA and actin representative immunoblot to validate the HA-tag incorporation in HeLa cells (n=3). (**b**) HA and MFN1 representative immunoblot of the immunoprecipitation in HeLa MFN1-HA cells (n=3). (**c**) FLAG representative immunoblot and

Ponceau staining in input, supernatant (SN) and eluate (IP) fractions of FLAG-immunoprecipitation in HeLa WT cells with or without transfection of the FLAG-RAB5C vector (n=3) (SN, supernatant). (**d**) Mfn2 and Rab5C representative immunoblot and band quantification in Scr and Mfn1-deficient myoblasts (n=6). (**e**) *Rab5C* and *Mfn2* mRNA levels upon transfection of siCtrl and siRab5C or siMfn2, respectively, in Scr or Mfn1-myoblasts (n=5-7). (**f**) Representative immunostainings of dsDNA (green) with nuclear subtraction of the signal (mtDNA) and EEA1 (red) in Scr and Mfn1-myoblasts upon siCtrl, siRab5C or siMfn2 transfection (Scale bar 10 µm in MERGE and 3 µm in INSET) (n=20 images per condition). Arrows point positive co-distribution. (**g**) Proposed working model for the mechanism described. (**d**) Two-sided Students' T-test, (**e**) Two-way ANOVA test and post-hoc t tests. Data are expressed as mean of n independent experiments ± SEM. *p vs Scr <0.05 in (**e**). *p vs Scr + siCtrl <0.05 and #p vs Mfn1KD + siCtrl <0.05 in (**e**). (**g**) Created with BioRender.com. (**a - e**) Source data is provided in the Source Data File.

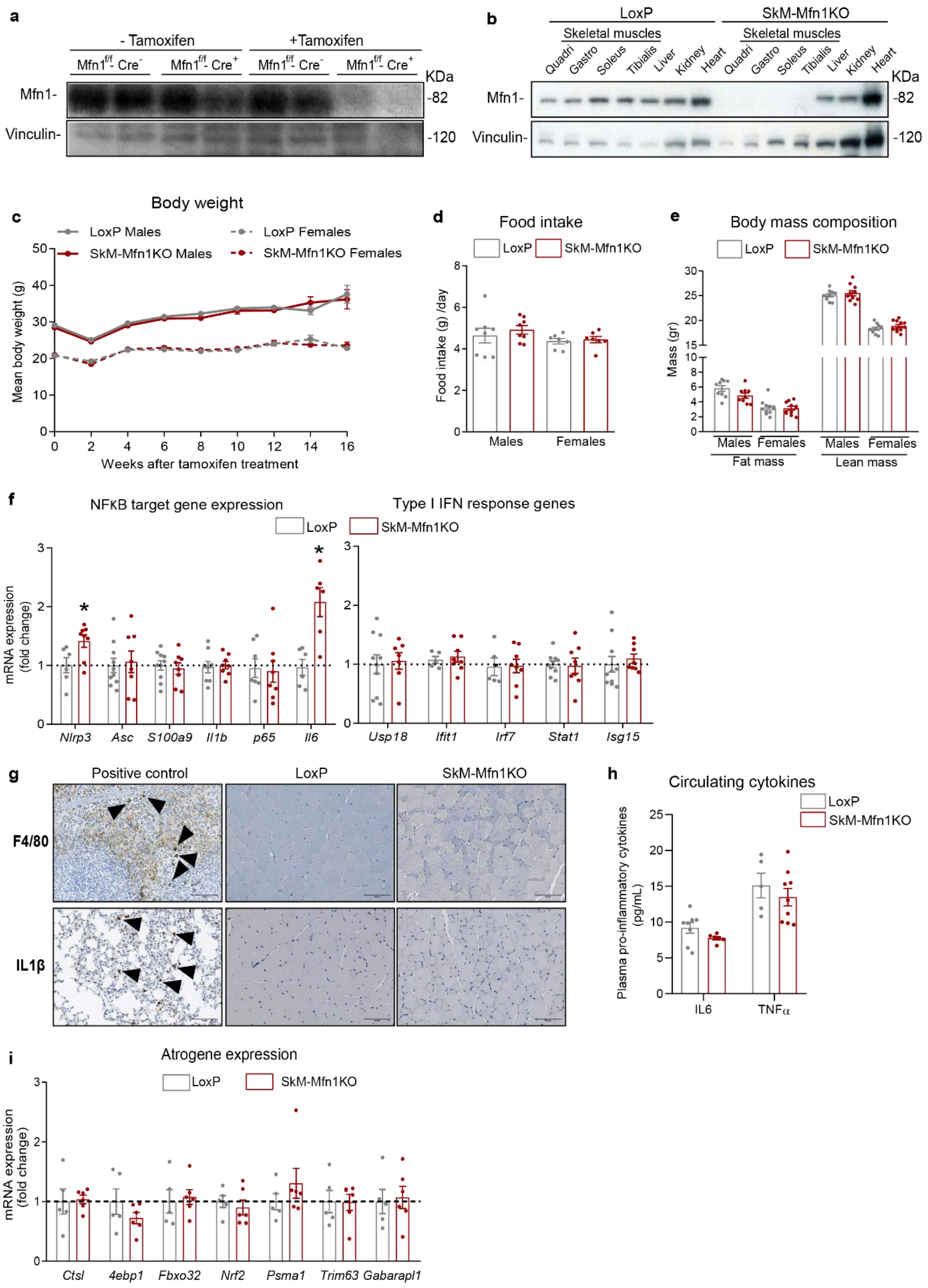

Supplementary Fig. 9 (**a**) Mfn1 and vinculin representative immunoblot of quadriceps muscles of Cre+ or Cre- animals with or without tamoxifen treatment (n=4). (**b**) Mfn1 and vinculin representative immunoblot in different tissues of LoxP and SkM-Mfn1KO mice (n=3).

(**c**) Body weight monitorization in male and female LoxP and SkM-Mfn1KO mice (n=20). (**d**) Food intake in male LoxP and SkM-Mfn1KO mice (n=8). (**e**) Body mass composition in male and female LoxP and SkM-Mfn1KO mice (n=9). (**f**) NFκB target and type I IFN response gene expression levels in quadriceps muscles of LoxP (n=6-9) and SkM-Mfn1KO female mice (n=6-10). (**g**) Representative immunohistochemistry (IHC) against F4/80 and IL1β in cross-sectional sections of gastrocnemius muscles of LoxP and SkM-Mfn1KO male mice (n=4). Black arrow point positive staining. Positive control in F4/80 IHC is an inflamed pancreas and in IL1β IHC is an inflamed lung. (**h**) Plasma levels of pro-inflammatory cytokines in LoxP (n=5-8) and SkM-Mfn1KO male mice (n=6-9). (**i**) Atrogene expression levels in quadriceps muscles of male LoxP (n=5) and SkM-Mfn1KO mice (n=5-6). (**c, e, f**) Two-way ANOVA test and post-hoc t tests, (**d, h**) Two-sided Students' T-test, (**f, i**) Two-sided Students' T-test per gene. Data are expressed as mean ± SEM. *p vs LoxP <0.05 in (**f**). (**a – f, h** and **i**) Source data is provided in the Source Data File.

Supplementary tables

Supplementary Table 1. Primary antibodies.

Antibody	Catalog no.	Application	Dilution
α-Tubulin	T5168 (Sigma-Aldrich)	WB	1:8000
β-Actin	A1978 (Sigma-Aldrich)	WB	1:5000
anti-Vinculin	Ab18058 (Abcam)	WB	1:5000
anti-Mfn1	SC-50330 (SantaCruz Biotechnology)	WB	1:500
anti-Mfn1	Kindly provided by Dr. Carles Cantó	WB	1:1000
anti-Mfn2	11925 (Cell Signaling)	WB	1:1000
anti-Drp1	611112 (BD transduction Lab)	WB	1:500
anti-Fis1	GTX111010 (GeneTex)	WB	1:1000
anti-LAMP1	SC-19992 (SantaCruz Biotechnology)	WB, IF	1:1000, 1:400
anti-TIMM23	SC-514463 (SantaCruz Biotechnology)	WB	1:1000
anti-YME1L	11510-1-AP (Protein Tech Group)	WB	1:500
anti-SLC25A33	TA309042 (Origene)	WB	1:500
anti-Rab5C	NBP1-80858 (Novus)	WB	1:1000
anti-FLAG	14793S (Cell Signaling)	WB	1:500
Anti-HA	3724S (Cell Signaling)	WB	1:1000
anti-F4/80	14-4801-85 (Clone BM, eBioscience)	IHC	1:100
anti-IL1β	Ab9722 (Abcam)	IHC	1:1000
anti-TOMM20	SC-17764 (SantaCruz Biotechnology)	IF	1:400
anti-TLR9	SC-52966 (SantaCruz Biotechnology)	IF	1:400
anti-cGAS	SC-515777 (SantaCruz Biotechnology)	IF	1:400
anti-dsDNA	Ab27156 (Abcam)	IF	1:400
Anti-Rab5	1673547S (Cell Signaling)	IF, IG	1:400, 1:4
Anti-EEA1	3288S (Cell Signaling)	IF	1:400
Anti-Rab7	9367 (Cell Signaling)	IF	1:400
Anti-HRS	15087 (Cell Signaling)	IF	1:400
Anti-SdhA	459200 (Life Technologies)	IG	1:20

Supplementary Table 2. pLKO.1 plasmids.

Targeted gene	Target sequence
Mfn1	CCCAGTGTACTGAAAGTGTAT
Mfn2	CAAGACTACAAGCTGCGAATT
Fis1	CCTGATTGATAAGGCCATGAA
Dnml1 (Drp1)	CGGTGGTGCTAGGATTTGTTA
Yme1L	CGAGCCAAACATATCTTGAAA

Supplementary Table 3. MISSION® siRNAs.

Targeted gene	MISSION® siRNA reference
Mfn1	SASI_Mm01_00037358
Mfn2	SASI_Mm01_00027321
Fis1	SASI_Mm01_00022625
Dnml1 (Drp1)	SASI_Mm01_00125378
Rab5C	SASI_Mm01_00105011
Vdac1	SASI_Mm02_00321251
Bax	SASI_Mm02_00311843
Ppid	SASI_Mm01_00039330
DnaseIIa	SASI_Mm01_00135573

Supplementary Table 4. Primers targeting mtDNA-encoded sequences.

Gene	Forward	Reverse
D-loop1	AATCTACCATCCTCCGTGAAACC	TCAGTTTAGCTACCCCCAAGTTTAA
D-loop3	TCCTCCGTGAAACCAACAA	AGCGAGAAGAGGGGCATT
Cytb	GCTTTCCACTTCATCTTACCATTTA	TGTTGGGTTGTTTGATCCTG
Cox2	GGAACACTCCAAAAACAGACCT	CCACCACTGGGTATTGAGTAGAA
16s	CACTGCCTGCCCAGTGA	ATACCGCGGCCGTTAAA
Nd4	AACGGATCCACAGCCGTA	AGTCCTCGGGCCATGATT

Supplementary Table 5. SYBR green primers.

Gene	Forward	Reverse
b-actin	GGTCATCACTATTGGCAACGA	GTCAGCAATGCCTGG
36b4	TCATCCAGCAGGTGTTTGACA	GGCACCGAGGCAACAGTT
Gapdh	AGGCCGGTGCTGAGTATGTC	TGCCTGCTTCACCACCTTCT
Mfn1	CCTACTGCTCCTTCTAACCCA	AGGGACGCCAATCCTGTGA
Mfn2	AGAACTGGACCCGGTTACCA	CACTTCGCTGATACCCCTGA
Dnml1	CGTGACAAATGAAATGGTGC	CATTAGCCCACAGGCATCAG
Fis1	TGTCCAAGAGCACGCAATTTG	CCTCGCACATACTTTAGAGCCTT
Rab5C	TGGTCCTCCGCTTTGTCAAG	TGACCGTTGTATCGTCTAAGCA
Vdac1	CCCACATACGCCGATCTTGG	GTGGTTTCCGTGTTGGCAGA
Bax	TGAAGACAGGGGCCTTTTTG	AATTCGCCGGAGACACTCG
Ppid	AACCCGCGAGTCTTCTTTGAC	TAATTCGGTGGAAAGGGCATC
DnaseIIa	AAGCCCTGAGCTGCTATGG	ATACGTCAGTCCCTTTGGAGTA
Nlrp3	CGAGACCTCTGGGAAAAAGCT	CATACCATAGAGGAATGTGATGTACA
Asc	GAAGCTGCTGACAGTGCAAC	GCCACAGCTCCAGA CTCTTC
S100a9	TGAGCAAGAAGGAATTCAGACAAA	TGTGTCCAGGTCCTCCATGA
Rage	GAAGGCTCTGTGGGTGAGTC	CCGCTTCCTCTGACTGATTC
Hmgb1	CGCGGAGGAAAATCAACTAA	TCATAACGAGCCTTGTCAGC
Myd88	GAAACTCCACAGGCGAGCGTA	GTTAAGCGCGACCAAGGGTATG
Tnfa	CACAAGATGCTGGGACAGTGA	TCCTTGATGGTGGTGCATGA
Il6	GCCCACCAAGAACGATAGTCA	CAAGAAGGCAACTGGATGGAA
Il1b	GCAACTGTTCCTGAACTCAACT	ATCTTTTGGGGTCCGTCAACT
RelA (p65)	GACCAACAATAACCCCTTTCAC	GTTTGAGATCTGCCCTGATGG
Usp18	AGAGTTAGCAAGCTCCGACAT	TGAGGTGAATGGTCAAGGTTTG
Ifit1	CTGAGATGTCACTTCACATGGAA	GTGCATCCCCAATGGGTTCT
Irf7	CAATTCAGGGGATCCAGTTG	AGCATTGCTGAGGCTCACTT
Stat1	CGCGCATGCAACTGGCATATAACT	ATGCTTCCGTTCCCACGTAGACTT
Isg15	GGTGTCCGTGACTAACTCCAT	TGGAAAGGGTAAGACCGTCCT
Ifnb	CCCTATGGAGATGACGGAGA	CCCAGTGCTGGAGAAATTGT
Ctsl	GTGGACTGTTCTCACGCTCAAG	TCCGTCCTTCGCTTCATAGG
4ebp1	CACGCTCTTCAGCACCAC	GGAGGCTCATCGCTGGTAG
Fbxo32	GCAAACACTGCCACATTCTCTC	CTTGAGGGGAAAGTGAGACG
Gabarapl1	CATCGTGGAGAAGGCTCTA	ATACAGCTGGCCCATGGTAG
Nrf2	GCAACTCCAGAAGGAACAGG	AGGCATCTTGTTTGGGAATG
Psma1	CATTGGAATCGTTGGTAAAGAC	GTTCATCGGCTTTTTCTGC
Trim63	TGTCTGGAGGTCGTTTCCG	ATGCCGGTCCATGATCACTT
Fgf21	ATGGAATGGATGAGATCTAGAGTTGG	TCTTGGTGGTCATCTGTGTAGAGG

Gating strategies for FACS analyses

Mitotracker Green (ThermoFisher): Scr C2C12

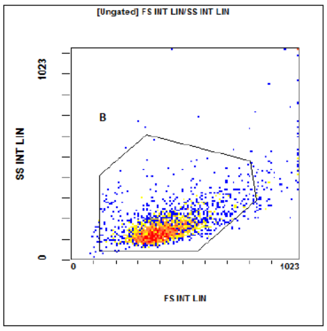

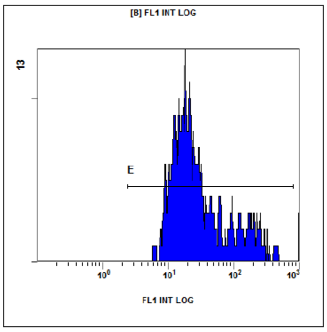

[Ungated] FS INT LIN/SS INT LIN					
Region	Number	%Total	%Gated	X-Mean	Y-Mean
ALL	2685	100.00	100.00	435	164
B	2562	95.42	95.42	418	152

[B] FL1 INT LOG					
Region	Number	%Total	%Gated	X-Mean	Y-Mean
ALL	2562	95.42	100.00	88.9	###
E	2529	94.19	98.71	78.1	###

Mitotracker Deep Red (ThermoFisher): Scr C2C12

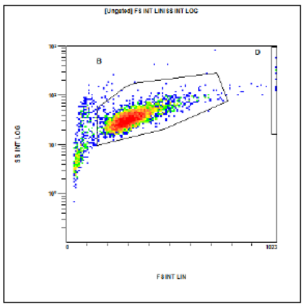

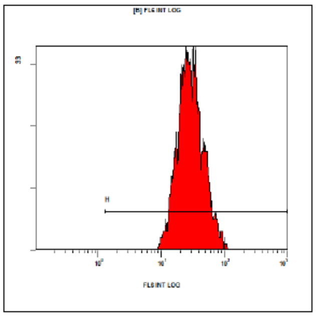

[Ungated] FS INT LIN/SS INT LOG					
Region	Number	%Total	%Gated	X-Mean	Y-Mean
ALL	5000	100.00	100.00	298	36.9
B	4499	89.98	89.98	318	37.2
D	9	0.18	0.18	1.02e+003	286

[B] FL6 INT LOG					
Region	Number	%Total	%Gated	X-Mean	Y-Mean
ALL	4499	89.98	100.00	34.5	###
H	4497	89.94	99.96	34.5	###

TMRE (ThermoFisher): Scr C2C12

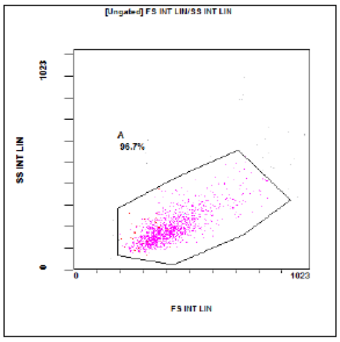

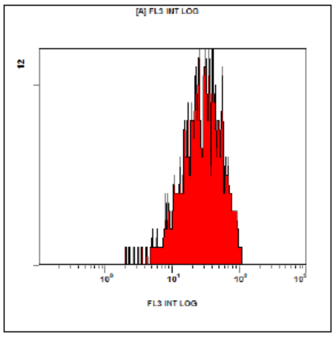

[Ungated] FS INT LIN/SS INT LIN			
Region	Number	%Gated	X-Mean
ALL	2662	100.00	428
A	2573	96.66	415

[A] FL3 INT LOG			
Region	Number	%Gated	X-Mean
ALL	2573	100.00	32.4

MitoSOX (ThermoFisher): Scr C2C12

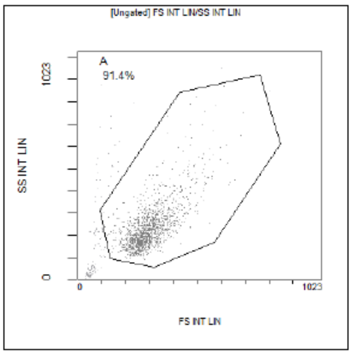

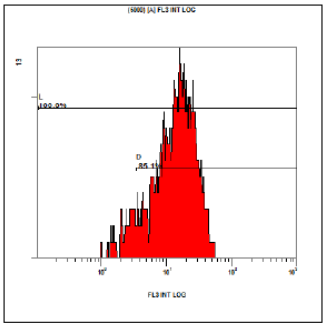

[Ungated] FS INT LIN/SS INT LIN

Region	Number	%Gated	X-Mean
ALL	3051	100.00	288
A	2788	91.38	306

[A
Re
AI
HI
H:

(5000) [A] FL3 INT LOG

Region	Number	%Gated	X-Mean
ALL	2788	100.00	15.8
D	2372	85.08	18.2
L	2788	100.00	15.8
