# Supplementary material for: Disruption of mitochondrial dynamics triggers muscle inflammation through interorganellar contacts and mitochondrial DNA mislocation
Source: Nat Commun. 2023 Jan 6;14:108. doi: 10.1038/s41467-022-35732-1 (PMC9822926; doi:10.1038/s41467-022-35732-1)
Supplement: Supplementary file 2 — Reporting Summary [file 41467_2022_35732_MOESM2_ESM.pdf]

nature portfolio

Corresponding author(s): David Sebastián and Antonio Zorzano

Last updated by author(s): Nov 21, 2022

Reporting Summary

Nature Portfolio wishes to improve the reproducibility of the work that we publish. This form provides structure for consistency and transparency in reporting. For further information on Nature Portfolio policies, see our Editorial Policies and the Editorial Policy Checklist.

Statistics

For all statistical analyses, confirm that the following items are present in the figure legend, table legend, main text, or Methods section.

n/a	Confirmed	
☐	☒	The exact sample size (*n*) for each experimental group/condition, given as a discrete number and unit of measurement
☐	☒	A statement on whether measurements were taken from distinct samples or whether the same sample was measured repeatedly
☐	☒	The statistical test(s) used AND whether they are one- or two-sided<br>*Only common tests should be described solely by name; describe more complex techniques in the Methods section.*
☒	☐	A description of all covariates tested
☒	☐	A description of any assumptions or corrections, such as tests of normality and adjustment for multiple comparisons
☐	☒	A full description of the statistical parameters including central tendency (e.g. means) or other basic estimates (e.g. regression coefficient) AND variation (e.g. standard deviation) or associated estimates of uncertainty (e.g. confidence intervals)
☐	☒	For null hypothesis testing, the test statistic (e.g. F, t, r) with confidence intervals, effect sizes, degrees of freedom and P value noted<br>*Give P values as exact values whenever suitable.*
☒	☐	For Bayesian analysis, information on the choice of priors and Markov chain Monte Carlo settings
☒	☐	For hierarchical and complex designs, identification of the appropriate level for tests and full reporting of outcomes
☐	☒	Estimates of effect sizes (e.g. Cohen's *d*, Pearson's *r*), indicating how they were calculated

Our web collection on statistics for biologists contains articles on many of the points above.

Software and code

Policy information about availability of computer code

Data collection	Data collection was achieved using Excel 2016. For qPCR data collection QuantStudio 6 software was used.
Data analysis	Data analysis was performed using GraphPad Prism version 8.0.1 and 9.4.1 for Windows. Image processing and quantification were performed using Image J 1.53c with JACoP and MiNA plugins. For proteomic studies, Proteome Discoverer software v2.3 (Thermo) using Sequest HT search engine, SwissProt Human release 2019_01 and Contaminants database, Sequest HT, Andromeda, and SAINTexpress-spc v3.11 softwares were used.

For manuscripts utilizing custom algorithms or software that are central to the research but not yet described in published literature, software must be made available to editors and reviewers. We strongly encourage code deposition in a community repository (e.g. GitHub). See the Nature Portfolio guidelines for submitting code & software for further information.

Data

Policy information about availability of data

All manuscripts must include a data availability statement. This statement should provide the following information, where applicable:

- Accession codes, unique identifiers, or web links for publicly available datasets
- A description of any restrictions on data availability
- For clinical datasets or third party data, please ensure that the statement adheres to our policy

All data generated or analysed during this study are included in this published article (and its supplementary information files). Databases used include: SwissProt Human release 2019_01 and Contaminants databases.

Human research participants

Policy information about studies involving human research participants and Sex and Gender in Research.

Reporting on sex and gender	N/A
Population characteristics	N/A
Recruitment	N/A
Ethics oversight	N/A

Note that full information on the approval of the study protocol must also be provided in the manuscript.

Field-specific reporting

Please select the one below that is the best fit for your research. If you are not sure, read the appropriate sections before making your selection.

☒ Life sciences ☐ Behavioural & social sciences ☐ Ecological, evolutionary & environmental sciences

For a reference copy of the document with all sections, see nature.com/documents/nr-reporting-summary-flat.pdf

Life sciences study design

All studies must disclose on these points even when the disclosure is negative.

Sample size	We selected a sample size of 6-7 independent observations (as minimum) in mouse studies in order to be able to detect statistical differences with a statistical power values of 80%. This was assuming that we would like to detect differences of 25% of in the means of control and experimental groups and that taking into account that the SEM is around 10% of mean values.
Data exclusions	Given a high variability in the expression levels of inflammatory genes, some data from gene expression analyses had to be excluded based on outlier identification (GraphPad Prism). To assure reproducibility and robustness of the results, these analyses were repeated. Only healthy mice were used in the study. In case, mice showed clearl signs of sickness, they were excluded from the study.
Replication	Reproducibility was achieved in all the experiments included in this study. In mouse studies, at least 2 different cohorts were used and the same results were obtained in all of them. In cell culture experiments, at least 3 independent experiments were performed.
Randomization	Mice were randomly assigned to experimental groups according to their date of birth, designing the groups with an even distribution of mice with different genotypes. For cell cultured experiments samples were randomized based on the expression of the protein knocked-down (control group and KD group).
Blinding	The investigators were blinded in all studies involving mice and analysis of imaging studies in cells. This was achieved by the use of a simple coding system of samples that enable their tracking while the investigators were blinded.

Reporting for specific materials, systems and methods

We require information from authors about some types of materials, experimental systems and methods used in many studies. Here, indicate whether each material, system or method listed is relevant to your study. If you are not sure if a list item applies to your research, read the appropriate section before selecting a response.

Materials & experimental systems

n/a	Involved in the study	
☐	☒	Antibodies
☐	☒	Eukaryotic cell lines
☒	☐	Palaeontology and archaeology
☐	☒	Animals and other organisms
☒	☐	Clinical data
☒	☐	Dual use research of concern

Methods

n/a	Involved in the study	
☒	☐	ChIP-seq
☐	☒	Flow cytometry
☒	☐	MRI-based neuroimaging

Antibodies

Antibodies used	All the primary antibodies used are described in Table S1 (Name, catalog number, supplier and dilution used) and include: α-Tubulin T5168 (Sigma-Aldrich) 1:8000; β-Actin A1978 (Sigma-Aldrich) WB 1:5000; anti-Vinculin Ab18058 (Abcam) WB 1:5000; anti-MFN1 SC-50330 (SantaCruz Biotechnology) WB 1:500; anti-MFN1 generated and provided by Dr. Carles Cantó WB 1:1000; anti-MFN2 11925 (Cell Signaling) WB 1:1000; anti-DRP1 611112 (BD transduction Lab) WB 1:500; anti-FIS1 GTX111010 (GeneTex) WB 1:1000; anti-LAMP1 SC-19992 (SantaCruz Biotechnology) WB, IF 1:1000, 1:400; anti-TIMM23 SC-514463 (SantaCruz Biotechnology) WB 1:1000 anti-YME1L 11510-1-AP (Protein Tech Group) WB 1:500; anti-SLC25A33 TA309042 (Origene) WB 1:500; anti-RAB5C NBP1-80858 (Novus) WB 1:1000; anti-FLAG 14793S (Cell Signaling) WB 1:500; Anti-HA 3724S (Cell Signaling) WB 1:1000; anti-F4/80 14-4801-85 (Clone BM, eBioscience) IHC 1:100;anti-IL1β Ab9722 (Abcam) IHC 1:1000; anti-TOMM20 SC-17764 (SantaCruz Biotechnology) IF 1:400; anti-TLR9 SC-52966 (SantaCruz Biotechnology) IF 1:400; anti-cGAS SC-515777 (SantaCruz Biotechnology) IF 1:400; anti-dsDNA Ab27156 (Abcam) IF 1:400; Anti-RAB5 1673547S (Cell Signaling) IF, IG 1:400, 1:4; Anti-EEA1 3288S (Cell Signaling) IF 1:400; Anti-RAB7 9367 (Cell Signaling) IF 1:400; Anti-HRS 15087 (Cell Signaling) IF 1:400; Anti-SDHA 459200 (Life Technologies) IG 1:20. Secondary antibodies used were: donkey anti-mouse HRP (715-035-150, Jackson Laboratories) and donkey anti-rabbit HRP (711-035-152, Jackson Laboratories), donkey anti-rat HRP (760-4457, Roche).
Validation	Validation of commercial antibodies was based on information provided by the manufacturer and/or previous use in our laboratory (Knock out validated).

Eukaryotic cell lines

Policy information about cell lines and Sex and Gender in Research

Cell line source(s)	C2C12 and HeLa cell lines
Authentication	C2C12 cells were acquired from ATCC (Catalog number CRL-1772). HeLa cells are routinely used in our laboratory.
Mycoplasma contamination	Mycoplasma contamination was evaluated every two weeks and only mycoplasma-free cells were used in all experiment. In the unfrequent event of finding mycoplasma contamination, cells were immediately discarded and new mycoplasma-free vials were thawed.
Commonly misidentified lines (See ICLAC register)	No commonly misidentified cell lines were used in this study.

Animals and other research organisms

Policy information about studies involving animals; ARRIVE guidelines recommended for reporting animal research, and Sex and Gender in Research

Laboratory animals	Inducible skeletal muscle-specific knock-out mice for Mfn1, generated by crossing Mfn1 flox/flox mice with mice carrying Cre-ER under the control of human skeletal actin tamoxifen-inducible promoter (HSA). All mice were 4-6 months-old and C57Bl6/J pure genetic background.
Wild animals	The study did not involve wild animals.
Reporting on sex	Studies were performed in both male and female, and this is stated in the manuscript.
Field-collected samples	The study did not involve samples collected from the field.
Ethics oversight	Institutional Animal Care and Use Committee of the Barcelona Science Park and University of Barcelona (Protocol number 9279, approved by the Department of Territory and Sustainability, General Directorate of Environmental Policies and the Natural Environment, Government of Catalonia).

Note that full information on the approval of the study protocol must also be provided in the manuscript.

Flow Cytometry

Plots

Confirm that:

- [x] The axis labels state the marker and fluorochrome used (e.g. CD4-FITC).
- [x] The axis scales are clearly visible. Include numbers along axes only for bottom left plot of group (a 'group' is an analysis of identical markers).
- [x] All plots are contour plots with outliers or pseudocolor plots.
- [x] A numerical value for number of cells or percentage (with statistics) is provided.

Methodology

Sample preparation	C2C12 were incubated with the appropiate dyes and rinsed in culture medium.
Instrument	Gallios
Software	Excel
Cell population abundance	10.000
Gating strategy	Alive cell population was selected by FCC/SCC values and then the fluorescence intensity of the studied fluorescent probe was recorded in the corresponding channel (FL1, FL3, FL6).

- [x] Tick this box to confirm that a figure exemplifying the gating strategy is provided in the Supplementary Information.
